# Targeting miR-27a/VE-cadherin interactions rescues cerebral cavernous malformations in mice

**DOI:** 10.1371/journal.pbio.3000734

**Published:** 2020-06-05

**Authors:** Jia Li, Yang Zhao, Jaesung Choi, Ka Ka Ting, Paul Coleman, Jinbiao Chen, Victoria C. Cogger, Li Wan, Zhongsong Shi, Thorleif Moller, Xiangjian Zheng, Mathew A. Vadas, Jennifer R. Gamble

**Affiliations:** 1 Centre for the Endothelium, Vascular Biology Program, Centenary Institute, The University of Sydney, Sydney, Australia; 2 Department of Biomedical Sciences, Faculty of Medicine, Health and Human Sciences, Macquarie University, Sydney, Australia; 3 Laboratory of Cardiovascular Signaling, Vascular Biology Program, Centenary Institute, The University of Sydney, Sydney, Australia; 4 Liver Injury and Cancer Program, Centenary Institute, The University of Sydney, Sydney, Australia; 5 Aging and Alzheimers Institute and ANZAC Research Institute and Concord Hospital, Charles Perkins Centre, The University of Sydney, Sydney, Australia; 6 Department of Neurosurgery, Sun Yat-Sen Memorial Hospital, Sun Yat-Sen University, Guangzhou, China; 7 Ranger Biotechnologies A/S, Arslev, Denmark; UCSD, UNITED STATES

## Abstract

Cerebral cavernous malformations (CCMs) are vascular lesions predominantly developing in the central nervous system (CNS), with no effective treatments other than surgery. Loss-of-function mutation in CCM1/krev interaction trapped 1 (KRIT1), CCM2, or CCM3/programmed cell death 10 (PDCD10) causes lesions that are characterized by abnormal vascular integrity. Vascular endothelial cadherin (VE-cadherin), a major regulator of endothelial cell (EC) junctional integrity is strongly disorganized in ECs lining the CCM lesions. We report here that microRNA-27a (miR-27a), a negative regulator of VE-cadherin, is elevated in ECs isolated from mouse brains developing early CCM lesions and in cultured ECs with CCM1 or CCM2 depletion. Furthermore, we show miR-27a acts downstream of kruppel-like factor (KLF)2 and KLF4, two known key transcription factors involved in CCM lesion development. Using CD5-2 (a target site blocker [TSB]) to prevent the miR-27a/VE-cadherin mRNA interaction, we present a potential therapy to increase VE-cadherin expression and thus rescue the abnormal vascular integrity. In CCM1- or CCM2-depleted ECs, CD5-2 reduces monolayer permeability, and in *Ccm1* heterozygous mice, it restores dermal vessel barrier function. In a neonatal mouse model of CCM disease, CD5-2 normalizes vasculature and reduces vascular leakage in the lesions, inhibits the development of large lesions, and significantly reduces the size of established lesions in the hindbrain. Furthermore, CD5-2 limits the accumulation of inflammatory cells in the lesion area. Our work has established that VE-cadherin is a potential therapeutic target for normalization of the vasculature and highlights that targeting miR-27a/VE-cadherin interaction by CD5-2 is a potential novel therapy for the devastating disease, CCM.

## Abstract 2

Cerebral cavernous malformation (CCM) is a disease for which, hitherto, surgery has been the only option. This study shows that a potential therapeutic, CD5-2, inhibits lesion development and vascular leak in the brains of CCM neonatal mice by targeting the endothelial cell–specific adhesion molecule VE-cadherin and restoring the vascular integrity of CCM lesions.

## Introduction

Cerebral cavernous malformations (CCMs) are vascular malformations mostly occurring in the central nervous system (CNS) [1]. CCM lesions are formed by abnormal dilated blood capillaries. One of the key features of CCM is the existence of gaps between endothelial cells (ECs) lining the lesions [2], which are leaky and can cause hemorrhagic stroke and seizure [3–5]. Currently, there is no treatment for CCMs other than surgery, which is limited by size and depth of the lesions.

Familial CCMs in humans are a result of loss-of-function mutation in either one of three genes, *CCM1* (krev interaction trapped 1 [*KRIT1*]), *CCM2*, and *CCM3* (programmed cell death 10 [*PDCD10*]) [6]. Mice with postnatal loss of *Ccm* genes in ECs develop similar brain vascular lesions as seen in human CCM patients. In both humans and mice, loss of CCM1 or CCM2 leads to similar defects in vascular integrity, morphology, and burden of CCM lesions, with CCM3 deletion giving a more severe phenotype [7–10].

The molecular pathways involved in CCM development include the kruppel-like factor (*KLF*)*2/4* pathway, endothelial-to-mesenchymal transition, RhoA/ROCK/phospho myosin light chain (pMLC), and, more recently, toll-like receptor 4 (TLR4) signaling through gut microbiome composition [3,9,11–14]. Although the clinical course of CCM disease is highly variable, a common feature is the disruptive EC–EC junctions and enhanced permeability [2,4,11,15], which likely explains the hemorrhage and inflammatory response in CCMs. Consistent with this, key endothelial junction molecules, including vascular endothelial cadherin (VE-cadherin), zonula occludens-1 (ZO-1), and claudin-5, are decreased or disorganized in lesions [10,16]. VE-cadherin, the ‘hub’ of EC junctional molecules, regulates the actin cytoskeleton through RhoA [17]. It also functions upstream of claudin-5, a key protein in tight junctions in the CNS [18,19]. In addition, VE-cadherin is a critical endothelial regulator of transforming growth factor-β (TGF-β) signaling [20]. Thus, VE-cadherin serves as an appealing target for CCM, since RhoA, claudin-5, and TGF-β are all deregulated in this disease [9,11,12,21] and are linked to disruption of vascular integrity. Therapies that increase the expression of VE-cadherin could restore vascular integrity and have a profound impact on CCM disease [22]. Indeed, the RhoA kinase inhibitors fasudil and, to a lesser extent, simvastatin decreased CCM lesions through restoring vascular integrity in mice [9,23].

MicroRNAs are key regulators of gene expression and play important roles in regulation of vascular integrity [24]. We previously showed microRNA-27a (miR-27a) targets VE-cadherin to disrupt vascular integrity [25,26]. Herein, we show miR-27a is overexpressed in brain ECs isolated from mice with CCMs. CD5-2 is a 15-nt-long target site blocker (TSB), which binds to the miR-27a binding site in the VE-cadherin 3′ UTR [25,26]. We previously showed CD5-2 increases endogenous VE-cadherin expression and restores vascular integrity in retinopathy, in peripheral ischemic vessels, and cancer vessels [25–27]. Based on this knowledge, we propose a strategy to treat CCMs through restoring VE-cadherin expression. We demonstrate here that the miR-27a/VE-cadherin interaction is CCM relevant and can be targeted by CD5-2 to normalize the vasculature of CCM lesions, resulting in decreased inflammation and inhibition of CCM pathologies. These data highlight VE-cadherin as a druggable target in diseases with abnormal vascular integrity.

## Results

### Up-regulation of microRNA-27a in the context of abnormal VE-cadherin expression in CCM pathology

**Fig 1:**
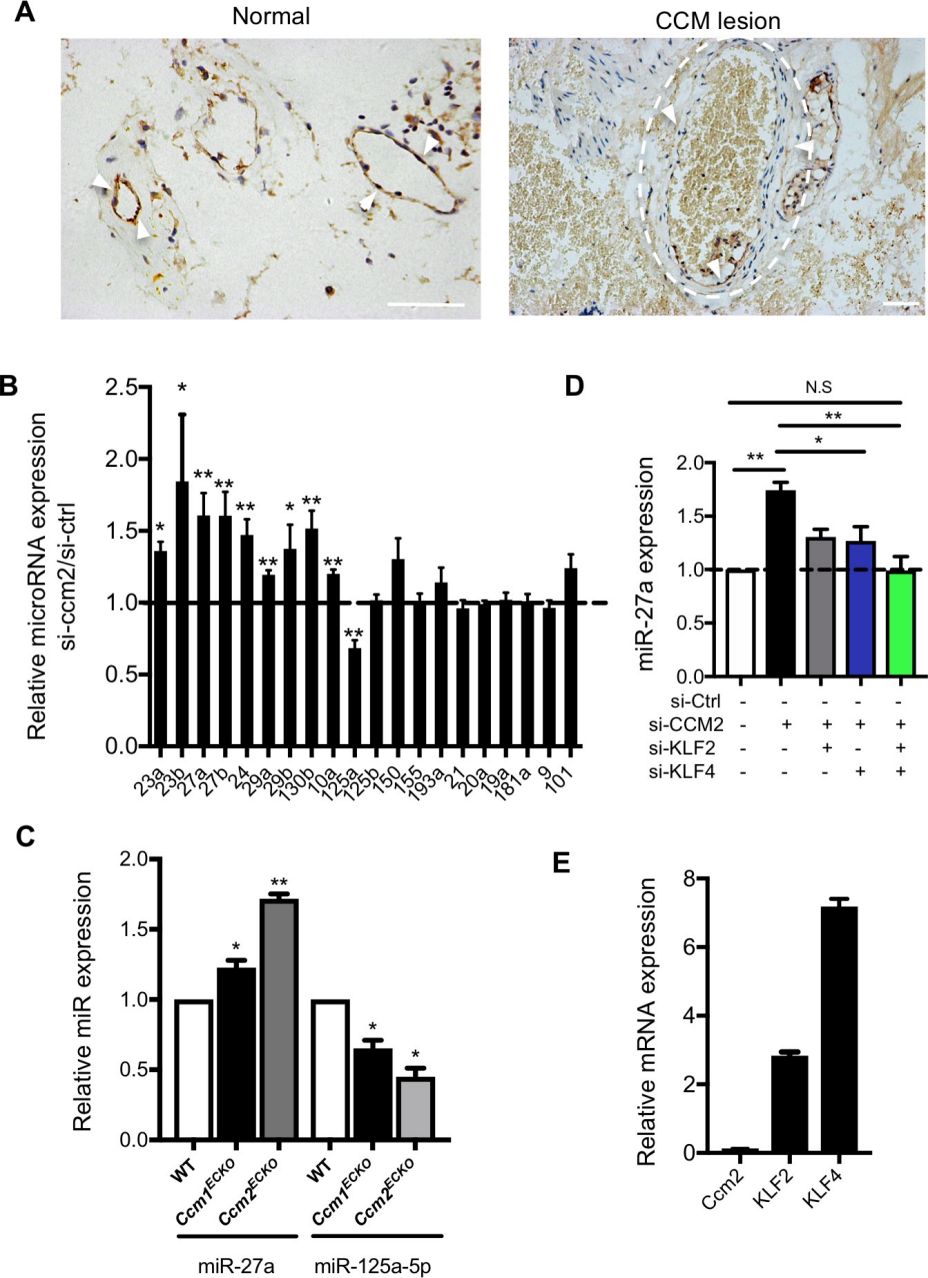
VE-cadherin and miR-27a are down-regulated and up-regulated in CCMs, respectively. (A) Representative immunohistochemistry staining of VE-cadherin of human lesion-free and CCM brain tissue. Arrowheads, VE-cadherin; dashed line, vascular lumen of CCM lesions. Bar, 100 μm (*n* = 6). (B) Real-time PCR measurement of microRNA expression in HUVECs treated with siRNA negative control (si-Ctrl) or siRNA to CCM2 (*n* = 3–4). (C) miR-27a was up-regulated and miR-125 was down-regulated in ECs isolated at P8 mice. RNA were from two separate preparations. The first includes the following: WT (*n* = 5), *Ccm1*^*ECKO*^ (*n* = 3), and *Ccm2*^*ECKO*^ (*n* = 2) mice. The second includes the following: WT (*n* = 4), *Ccm1*^*ECKO*^ (*n* = 3), and *Ccm2*^*ECKO*^ (*n* = 2) mice. (D) siRNA knockdown of KLF2 or KLF4 or both in HUVECs blocks loss-of-CCM2–induced expression of miR-27a (*n* = 3–4). (E) Measurement of KLF2 and KLF4 in VE-cadherin null ECs with CCM2 depleted by siRNAs (*n* = 3). Data represent mean ± SEM; *P < 0.05, **P < 0.01 determined by one-way ANOVA with Tukey correction. For the raw data used for quantification, see Fig 1 in S1 Data. CCM, cerebral cavernous malformation; EC, endothelial cell; HUVEC, human umbilical vein endothelial cell; KLF2, kruppel-like factor 2; miR-27a, microRNA-27a; siRNA, small interfering RNA; VE-cadherin, vascular endothelial cadherin; WT, wild-type.

Disruption of EC junctions and abnormal vascular integrity are key features in CCM pathology [9,11]. In the lesions of human patients with CCM, VE-cadherin expression is disrupted compared with its expression in normal vessels (Fig 1A). In a neonatal mouse model of CCM disease (the EC specific *Ccm2* deleted model, *Ccm2*^*ECKO*^), numerous CCM lesions appeared in the eye (S1A Fig) and hindbrains (S1B Fig) [3,21]. Furthermore, depletion of CCM1 or CCM2 in both primary human umbilical vein endothelial cells (HUVECs) and brain microvascular EC line, hCMEC/D3, using small interfering RNA (siRNA), resulted in decreased mRNA expression of key junctional molecules such as VE-cadherin and claudin-5 (S2A and S2B Fig) [28], and disruption of EC junctions, as shown by VE-cadherin staining (S2C Fig).

MicroRNAs play an important role in the regulation of vascular integrity [29]. A number of microRNAs were significantly regulated in CCM2-depleted ECs (Fig 1B), including miR-27a and miR-125a. MiR-27a targets VE-cadherin, and its enhanced expression results in loss of barrier integrity and induction of vascular leak [26]. In contrast, miR-125a is required for brain EC barrier formation [30]. Brain ECs extracted from *Ccm1*^*ECKO*^ and *Ccm2*^*ECKO*^ mice at 8 days post birth (P8), when lesions are obvious, showed a small but significant increase in miR-27a but decrease in miR-125a (Fig 1C). Indeed, this level is similar to that seen in vitro with CCM2 depletion mediated by siRNAs (Fig 1D). Even at P5, a time prior to the appearance of CCM lesions, miR-27a shows a 25% increase in ECs from *Ccm2*^*ECKO*^ mice (S2D Fig) [3]. In line with our finding, a recent report shows miR-27a is the most up-regulated microRNA in the brain tissues from human CCM patients [31], suggesting a potential role of miR-27a in CCM pathology.

Excessive expression of KLF2 and KLF4 are causal factors in CCM formation [3,14,32]. There are at least 3 putative KLF2/4 binding sites in the promoter of miR-27a, with two of these conserved between human and mouse [33]. Knockdown of KLF2 or KLF4 by siRNA partially inhibited the CCM2-depletion–induced miR-27a up-regulation in cultured ECs. Knockdown of both KLF2 and KLF4 completely rescued miR-27a up-regulation (Fig 1D). In addition, induction of the KLF2/4 pathway is independent of VE-cadherin, because KLF2 and KLF4 are both up-regulated, with loss of CCM2 in VE-cadherin null ECs (Fig 1E). These data implicate the presence of a regulatory pathway whereby loss-of-CCM up-regulates KLF2/4, resulting in increased miR-27a, which may decrease VE-cadherin expression.

### A TSB to miR-27a/VE-cadherin inhibits CCM pathogenesis

The data above suggest that blocking or preventing the negative regulation of miR-27a on VE-cadherin may restore vascular integrity in CCM lesions and be beneficial for the treatment of this disease. Because miR-27a has multiple targets besides VE-cadherin [34], targeting the miR-27a itself raises the potential of off-targets effects [35]. TSB with enhanced stability and specificity have been developed, which overcome this drawback by preventing the microRNA from gaining access to the binding site on the 3′ UTR of a specific target [36–44]. We developed CD5-2, a TSB that blocks the binding of miR-27a to its binding site specifically on VE-cadherin 3′ UTR (S3A Fig), leading to increased endogenous VE-cadherin expression, and regulates VE-cadherin–related pathways [25–27].

**Fig 2:**
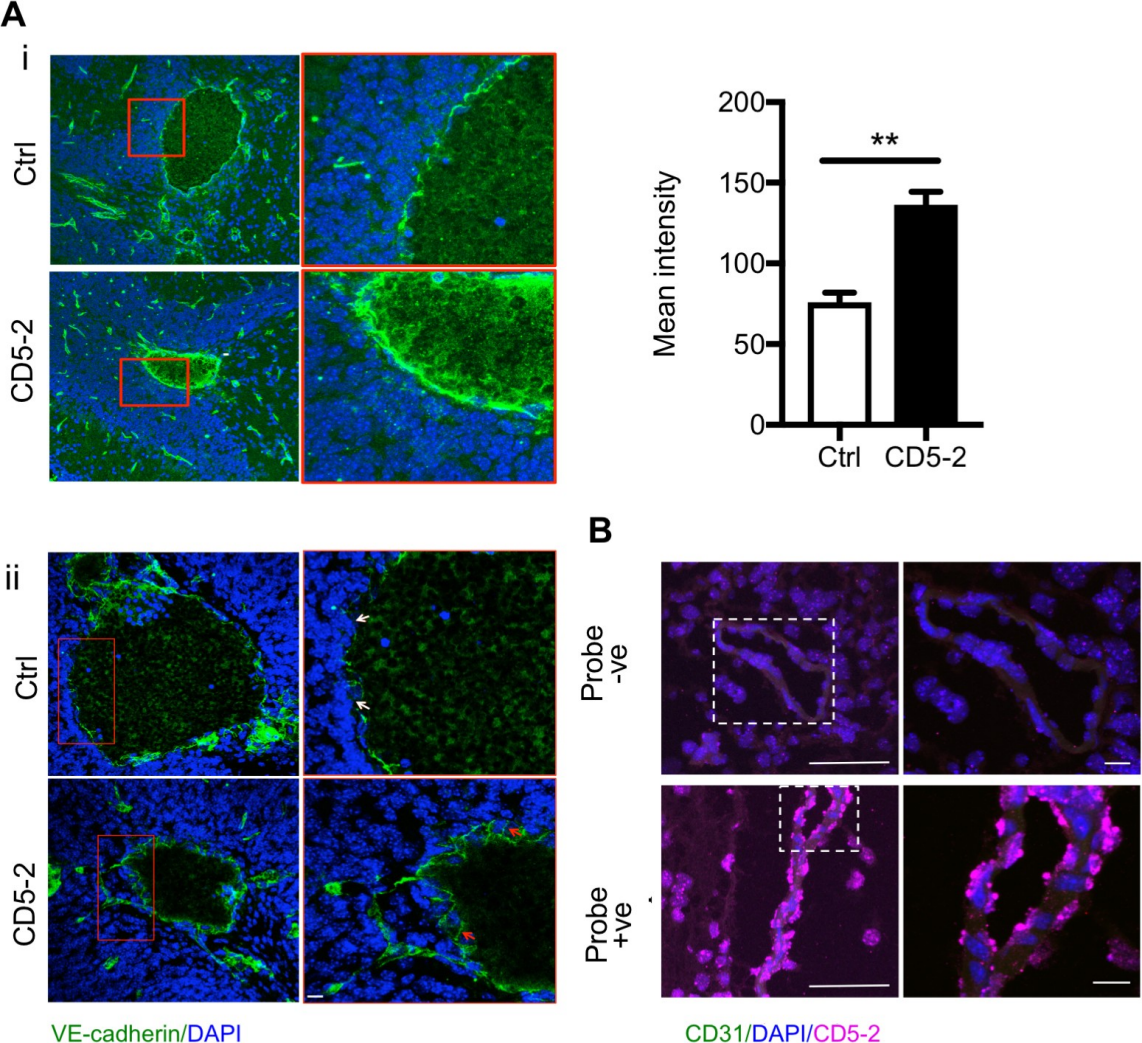
CD5-2, a TSB to miR-27a/VE-cadherin, can be delivered to brain vasculature and regulates VE-cadherin. (A) (i) CD5-2 increased VE-cadherin expression in CCM lesions from mouse samples given the control drug (Ctrl) or CD5-2. (ii) CD5-2 improves VE-cadherin localization in CCM lesions compared with mice given the control drug (Ctrl). In Ctrl, the white arrows point to disruptive VE-cadherin staining. In CD5-2, the red arrows point to junctional VE-cadherin. (B) In situ detection of CD5-2 in the blood vessels in the hindbrain of P6 *Ccm2*^*ECKO*^ animal, 6 hours after IP injection of CD5-2. The image without CD5-2 probe was used as background control (top). CD5-2 was detected as the color of cyan (bottom) with ECs stained with CD31. Bar, 50 μm (left); 10 μm (right). Values are shown as mean ± SEM, **P < 0.01, determined by Student *t* test. For the raw data used for quantification, see Fig 2 in S1 Data. CCM, cerebral cavernous malformation; EC, endothelial cell; IP, intraperitoneal; miR-27a, microRNA-27a; TSB, target site blocker; VE-cadherin, vascular endothelial cadherin.

To explore the effect of CD5-2 in CCMs, we first confirmed CD5-2 was able to restore VE-cadherin in CCM pathology. In cultured ECs with CCM1 or CCM2 depletion, CD5-2 rescued the decreased expression of VE-cadherin and its key downstream target, claudin-5 [18] (S3B Fig). In the animals with CCMs, CD5-2 was given via intraperitoneal (IP) injection on P6, when the earliest lesions had just started to form (S4A Fig) [3], and again on P8. Consistent with the in vitro data, when assessed at P12 (S4A Fig), injection into *Ccm2*^*ECKO*^ mice resulted in a significant increase in VE-cadherin expression in the EC lining of some CCM lesions (Fig 2Ai), whereas other lesions show improved VE-cadherin localization in the junction area (Fig 2Aii). In situ assay confirmed that CD5-2 localized into the brain vessels within 6 hours after IP injection to the *Ccm2*^*ECKO*^ mice at P6 (Fig 2B). ZO-1 is another key tight junction molecule in the maintenance of brain vascular integrity. In normal brain vessels, ZO-1 localizes to the junctions (S4Bi Fig). Although CD5-2 increases ZO-1 expression levels, it is not seen in the junctional area (S4Bii Fig).

**Fig 3:**
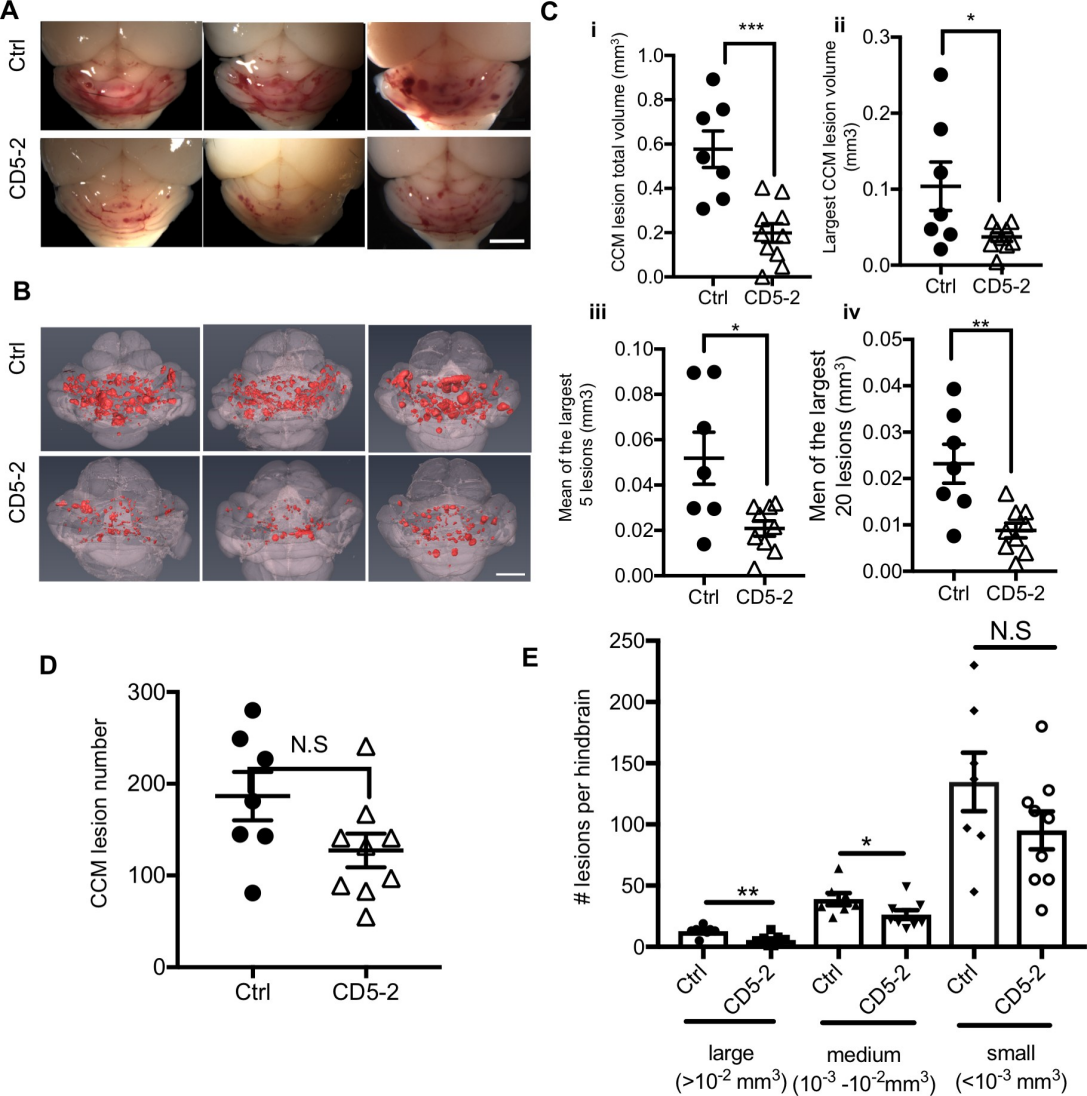
CD5-2 rescues CCM lesions in the hindbrain of *Ccm2*^*ECKO*^ mice. (A) Visual appearance of CCM lesions in the hindbrain of representative three P12 *Ccm2*^*ECKO*^ mice treated with control or CD5-2. Bar, 1 mm. (B) Composite microCT images of the same hindbrains shown in (A). Bar, 1 mm. CCM lesions are shown in red. (C) Quantitation of CCM lesion by (i) total lesion volume and (ii) the largest lesion volume, and (iii) the mean value of the largest 5 or (iv) 20 lesions. (D) Quantitation of lesion number. Ctrl, 186.6 ± 26.31; CD5-2, 127.3 ± 18.44. (E) Lesions were classified into three groups according to volume size (large: >10^−2^ mm^3^; medium: 10^−3^–10^−2^ mm^3^; small: <10^−3^ mm^3^). Large, Ctrl: 12.9 ± 1.6; CD5-2: 5.6 ± 1.3; medium, Ctrl: 39 ± 4.9; CD5-2: 26.3 ± 3.5; small, Ctrl: 134.7 ± 24.0; CD5-2: 95.1 ± 15.4, *n* = 7 mice for control-treated group (Ctrl) and *n* = 9 mice for CD5-2–treated group. Mice are from 4 different litters. Values are shown as mean ± SEM, N.S, not significant, *P < 0.05, **P < 0.01, ***P < 0.001, determined by Student *t* test. For the raw data used for quantification, see Fig 3 in S1 Data. CCM, cerebral cavernous malformation; Ctrl, control; microCT, micro–computed tomography.

CD5-2 also significantly reduced the vascular lesion burden, as shown for 3 representative mice (Fig 3A). Lesion size was firstly assessed morphologically in hindbrain sections by hematoxylin–eosin (HE) staining and by CD31 staining (S4C and S4D Fig). To precisely quantitate CCM lesions, we imaged P12 hindbrains using contrast-enhanced, high-resolution X-ray micro–computed tomography (microCT) and measured lesion volumes using semiautomated software [3,8]. Treatment of *Ccm2*^*ECKO*^ mice with CD5-2 conferred a significant reduction (Fig 3B) with around 60% inhibition in both total CCM lesion volume and on the size of the large lesions (Fig 3Ci-iv). In addition, there was a trend for CD5-2 to reduce the total lesion numbers, although the difference was not statistically significant (Fig 3D). However, when the lesions were classified into three groups according to their size, CD5-2 significantly reduced the large (>10^−2^ mm^3^) and medium (10^−3^–10^−2^ mm^3^) lesions but not the small (<10^−3^ mm^3^) lesions (Fig 3E). Large lesions are more likely to have abnormal EC junctions, shown by discontinuous VE-cadherin staining (S1Bii Fig). The lesion size is correlated with vascular leak susceptibility and reflects the severity of CCM disease [21,45]. Of note, the larger CCM lesions in humans are those that are more prone to hemorrhage, which therefore are more likely to need surgical intervention [23].

**Fig 4:**
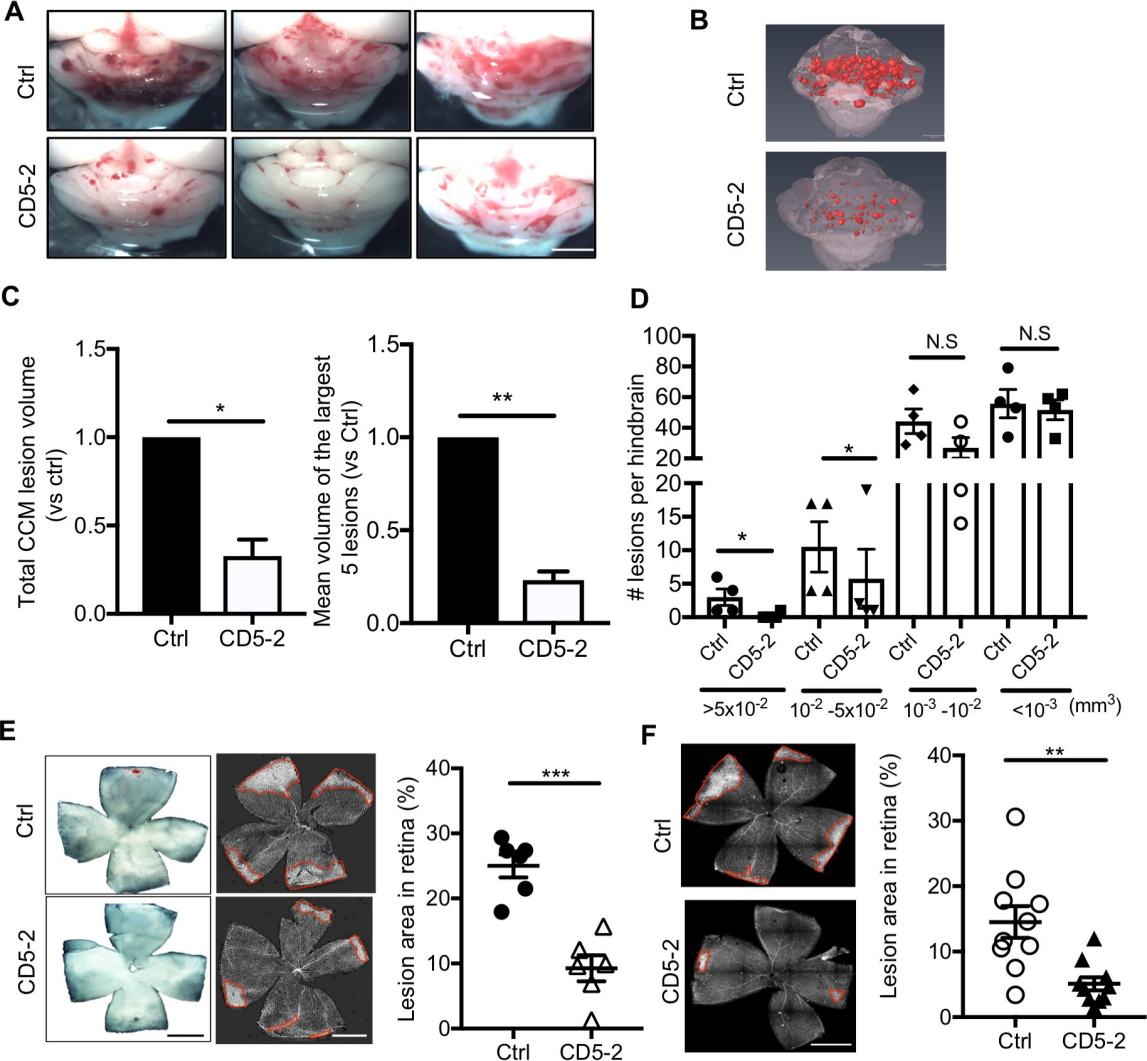
CD5-2 decreases established CCM lesions in the hindbrain of *Ccm2*^*ECKO*^ mice and lesions in the retina of *Ccm2*^*ECKO*^ mice. (A) Visual appearance of CCM lesions in the hindbrains of three representative P19 *Ccm2*^*ECKO*^ animals treated with control or CD5-2. Bar, 1 mm. (B) microCT images of the hindbrains from two mice shown in (A). CCM lesions are shown in red. (C) Quantitation of CCM lesion volume (normalized to 1.0 for mice that received control) by total lesion volume and mean size of the largest 5 lesions. (D) Number of lesions per brain within different size ranges. In (C-D), *n* = 4 mice for control-treated group and *n* = 4 mice for CD5-2–treated group. Mice are from 4 different litters. (E) Representative images of whole mount retina isolated from control- and CD5-2–treated littermates stained with isolectin-B4. Mice were treated at P6 and P8. Retinas were dissected at P12. Images are representative of 6 retinas from 3 mice (3 litters) for each treatment. Bar, 200 μm. Quantification is given as the percentage of the marked condensed vascular plexus at the leading edge against the total area of the retinal vasculature. (F) Mice were treated at P8 and P12. Retinas were dissected at P19. Images are representative of 10 retinas from 5 mice (3 litters) for each treatment. Bar, 200 μm. Values are shown as mean ± SEM. *P < 0.05, **P < 0.01, ***P < 0.001, determined by Student *t* test. For the raw data used for quantification, see Fig 4 in S1 Data. CCM, cerebral cavernous malformation; microCT, micro–computed tomography.

To further establish the therapeutic potential of CD5-2, we investigated whether it is able to affect established lesions—especially the large lesions. To address this possibility, we treated mice at P12 and P14 and brains were dissected and assessed at P19 (S5A Fig). This is the time frame when most of the CCM lesions are established with numerous large lesions, as is seen in Fig 4A [21]. CD5-2 was able to significantly reduce the volume of CCM lesions in the brain (Fig 4A and 4B). Although, as before, CD5-2 did not affect overall lesion numbers (S5B Fig), it significantly reduced the number and size of the very large, the large, and the medium sized lesions (Fig 4C and 4D).

In the retinas of *Ccm2*^*ECKO*^ mice, venous malformations developed at the periphery of vascular plexus, as visualized by isolectin-B4 staining (S1A Fig) and as previously reported [11,21]. In both early- (P6 and P8) and late-stage (P12 and P14) treated animals, CD5-2 inhibited the lesions in the retina (Fig 4E and 4F). Importantly, CD5-2–treated wild-type (WT) animals showed no noticeable differences in the brain and retina, or total hindbrain volume at P12 and P19 (S6A–S6C Fig). Overall, these results suggest that CD5-2 is able to increase VE-cadherin expression and rescue CCM lesion in mice with CCM disease.

### CD5-2 normalizes vasculature and blocks vascular leakage in CCM lesions

**Fig 5:**
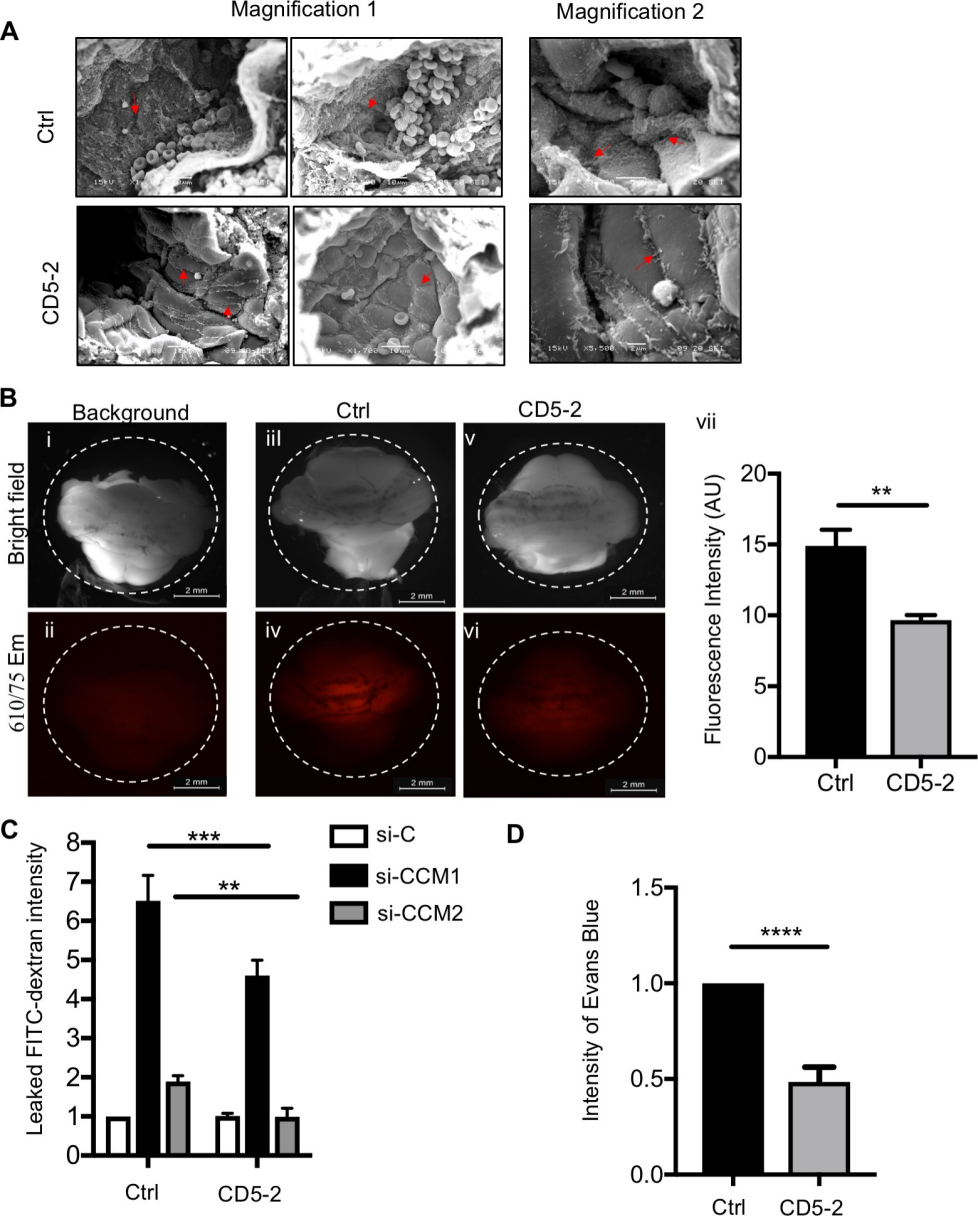
CD5-2 normalizes CCM lesion structure in *Ccm2*^*ECKO*^ mice. (A) Sample scanning electron micrographs of the blood vessels in hindbrain from mice that received Ctrl (top) and CD5-2 (bottom). Images were taken from multiple vessels. Two magnifications are shown: magnification 1, approximately 2,000×, bar: 10 μm; magnification 2, approximately 5,000×, top bar: 5 μm, bottom bar: 2 μm (EC junctions are indicated by red arrow). (B) Hindbrains photographed after fluorescent cadaverine injection. Bright-field (top) and fluorescence image with 555/60-nm excitation and 610/75-nm emission (bottom). An animal without cadaverin Alexa 555 injection was used as the background (i,ii); fluorescence from control (Ctrl) and CD5-2 treated animals was measured by stereomicroscope (iii-vi) and quantified by Fiji imageJ software (vii). Ctrl *n* = 5; CD5-2 *n* = 4 from 2 litters. Bar, 2 mm. (C) CCM1 or CCM2 in hCMEC/D3 was depleted by siRNAs. Cells were then transfected with control (Ctrl) or CD5-2. Endothelial monolayer permeability to FITC-dextran (40 KDa) was determined by measurement of absorbance at 490 nm (A490) (*n* = 2–3). (D) Miles assay of dermal permeability in heterozygous Ccm1 (*Tie2-Cre*, *Ccm1* ^*fl/+*^) mice in response to PBS or VEGF after pretreatment with control or CD5-2 (*n* = 6). Values are shown as mean ± SEM. **P < 0.01, ***P < 0.001, ****P < 0.0001, determined by Student t test or one-way ANOVA with Tukey correction. For the raw data used for quantification, see Fig 5 in S1 Data. CCM, cerebral cavernous malformation; EC, endothelial cell; FITC, fluorescein isothiocyanate; hCMEC/D3, human cerebral microvascular endothelial cell line; siRNA, small interfering RNA; VEGF, vascular endothelial growth factor.

VE-cadherin plays a central role in the regulation of vascular integrity. We next investigated whether CD5-2 altered the integrity of the endothelium in CCM pathology. Images taken by scanning electron microscope (SEM) show that CD5-2–treated animals have improved vascular integrity with clear EC junctions and a flattened morphology indicative of nonactivated cells and normalized endothelium (Fig 5A). Consistently, a single treatment of CD5-2 markedly prevented vascular leakage of the lesions, as measured by the amount of cadaverine that leaked from blood vessels into brain parenchyma (11) (Fig 5B). Notably, these in vivo findings are in line with the inhibition of fluorescein isothiocyanate (FITC)-dextran passing through the EC monolayer after CD5-2 treatment that we observed in ECs in vitro (Fig 5C), suggesting CD5-2 has a direct effect on improving EC junction. Furthermore, *Ccm1* heterozygous mice (*Tie2Cre*, *Ccm1*^*fl/+*^) exhibit increased basal vascular leak and stronger response to vascular endothelial growth factor (VEGF) [9]. CD5-2 restores the dermal vessel integrity under both basal conditions and after VEGF stimulation (Fig 5D and S7 Fig). These results suggested CD5-2 normalizes the vasculature in CCM pathology.

### CD5-2 reduces RhoA but does not regulate KLF2/4

Increased RhoA/ROCK activity and excessive KLF2/4 are both drivers for CCM development [12]. Further, RhoA/ROCK activation is linked with permeability regulation [46]. In the CCM1- or CCM2-depleted ECs, CD5-2 directly rescued RhoA activity and decreased its downstream pMLC, as visualized by immunofluorescence staining (S8A and S8B Fig). Although KLF2/4 overexpression is critical for CCM development [3,14], CD5-2 had no effect on the expression of KLF2/4, suggesting that its action may lie downstream of the induction of KLF2/4 (S8C Fig).

### CD5-2 directly rescues inflammatory response via VE-cadherin

**Fig 6:**
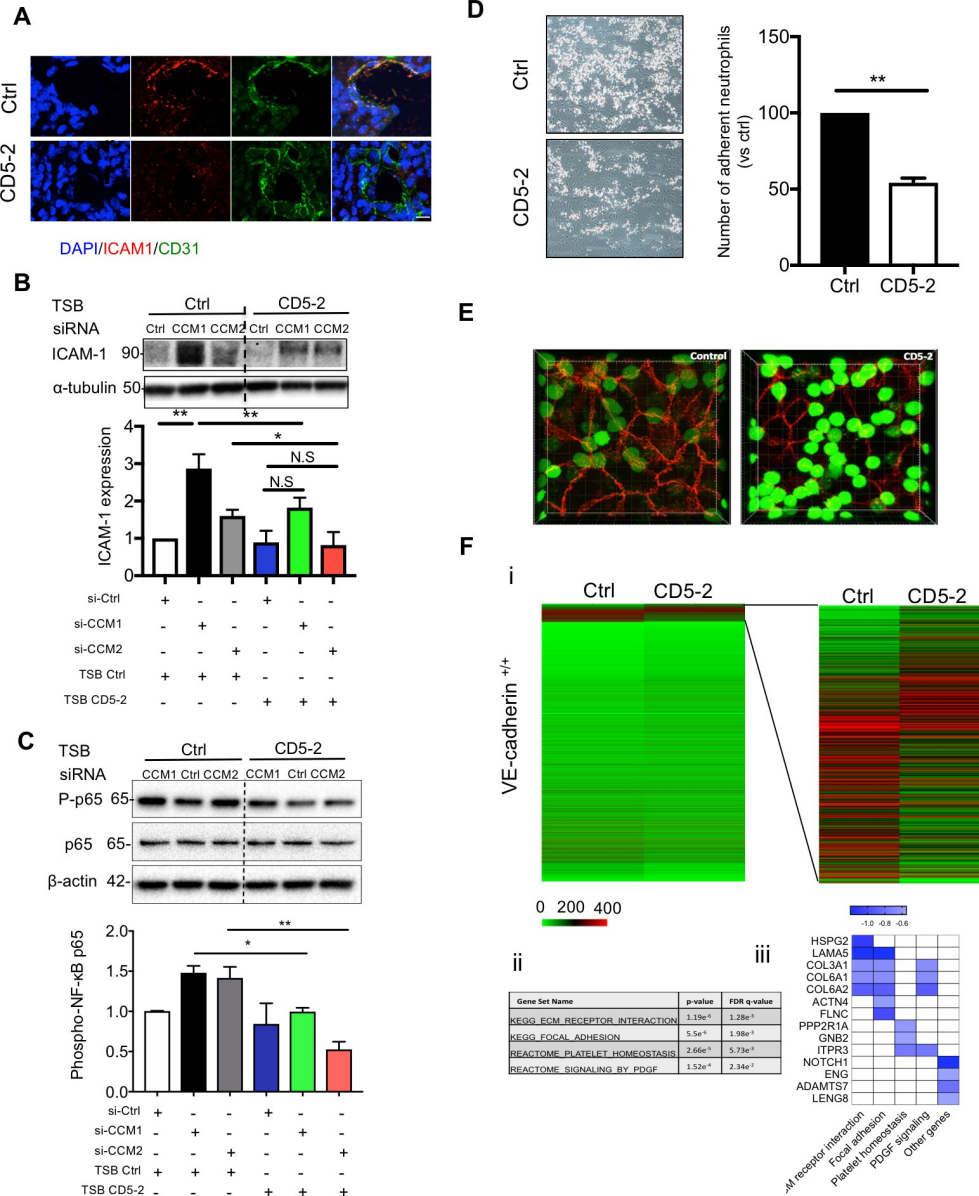
CD5-2 inhibits inflammatory response in CCM lesion via VE-cadherin. (A) CD5-2 effects on ICAM-1 expression in CCM lesion. Sections were triple-stained for DAPI (blue) or CD31 (green), ICAM-1 (red); 3 mice were utilized for each condition. Representative sections are shown. Bar, 8 μm. (B-C) Western blot analysis of ICAM-1 (B) and phospho-p65 (C) in brain microvascular ECs (hCMEC/D3) treated with 10 nM siRNAs for scramble control (Ctrl), CCM1, or CCM2 for 4 hours, followed by transfection of 15 nM CD5-2 or controls then cultured overnight. Molecular weights in kilodaltons are shown. ICAM-1 antibody (Cell signalling #4915) gave two bands (89 and 92 kDa). This antibody recognizes the ICAM-1 C-terminal portion, which is dense with ubiquitination and phosphorylation sites and, as such, #4915 can detect multiple sized ICAM moieties. Representative blots are shown (*n* = 3) with α-tubulin or β-actin rabbit used as loading control. (D) Representative images of dynamic adhesion of neutrophils to control- or CD5-2–treated ECs following the stimulation with TNF-α for 4 hours. Quantification of number of adherent neutrophils with control or CD5-2–treated ECs is given (*n* = 3). (E) Representative images of paracellular transmigration of neutrophils (green) through TNF-α–stimulated endothelium (VE-cadherin, red) in the presence of control and CD5-2 in vitro. Transmigrated neutrophils appear GFP^dim^. (F) (i) Heatmap of all genes between control (Ctrl) and CD5-2 (CD5-2) in VE-cadherin–expressing ECs (VE-cadherin^+/+^). (ii) GSEA analysis of differentially expressed genes (DEGs) between Ctrl- and CD5-2–treated ECs. (iii) Bar plot of selected inflammation-related DEGs inhibited by CD5-2. Values are shown as mean ± SEM. *P < 0.05, **P < 0.01, N.S, not significant, determined by Student *t* test or one-way ANOVA with Tukey correction. For the raw data used for quantification, see Fig 6 in S1 Data; Fig 6B and 6C in S1 Raw images. CCM, cerebral cavernous malformation; CD31, cluster of differentiation 31; EC, endothelial cell; GFP, green fluorescent protein; GSEA, Gene Set Enrichment Analysis; hCMEC/D3, human cerebral microvascular endothelial cell line; ICAM-1, intercellular adhesion molecule 1; NF-κB, nuclear factor kappa B; PDGF, platelet-derived growth factor; siRNA, small interfering RNA; TNF-α, tumor necrosis factor-α; TSB, target site blocker; VE-cadherin, vascular endothelial cadherin.

CCM lesions are also characterized by increased inflammation [11], which may contribute to the disruption in vascular integrity in CCM pathology [47]. In animals with CCMs, intercellular adhesion molecule 1 (ICAM-1) expression, a key adhesion molecule involved in neutrophil adhesion and transmigration, was inhibited in ECs lining the CCM lesions by the treatment of CD5-2 (Fig 6A) [11]. In addition, there were significantly less CD11b-expressing inflammatory cells associated with the vessel wall in the lesions of CD5-2–treated animals (S9A Fig) [11]. These results were consistent with the ability of CD5-2 to inhibit neutrophil migration into inflamed tissue in the MC38 colon carcinoma, in which CD5-2 inhibited the number of CD11b^+^/granulocyte (Gr)1^hi^ in the solid tumor tissue. In combination with anti–programmed cell death protein 1 (PD1), there was a further inhibition of the number of CD11b^+^/Gr1^hi^ cells in the tumor (S9B Fig).

To determine whether the action of CD5-2 is directly on the endothelium to inhibit inflammation, we turned to in vitro studies. CCM1 or CCM2 depletion up-regulated ICAM-1 expression and increased activity of NF-κB, an essential transcription factor involved in inflammation (Fig 6B and 6C) [48]. Treatment of the ECs with CD5-2 reversed these increases, suggesting a direct inhibition on the inflammatory response in ECs (Fig 6B and 6C) [11,49,50]. Furthermore, CD5-2 pretreatment of the endothelium inhibited both neutrophil adhesion and transmigration across the EC monolayer, two key steps involved in inflammation [51,52]. Using a model of neutrophil adhesion under flow (shear stress is 2 dyns/cm^2^), tumor necrosis factor-α (TNF-α) stimulation increased the number of adherent neutrophils in control-treated ECs, as expected [53]. In contrast, prior treatment of the ECs with CD5-2 resulted in a significant reduction in the number of adherent neutrophils (Fig 6D and S1 Movie). In addition, CD5-2 inhibited the transmigration of neutrophils across the endothelium, as ascertained by using a transwell migration assay (S9C Fig). To visualize the process of neutrophil transmigration in vitro, we used live cell fluorescent imaging. Neutrophils were labelled with CellTrack Green and allowed to transmigrate through TNF-α–stimulated ECs that had been treated either with control or CD5-2. As shown in S2 Movie and in Fig 6E, neutrophils preferentially transmigrated via the paracellular route, and CD5-2 significantly inhibited transmigration of neutrophils (transmigrated cells appear green fluorescent protein [GFP]^dim^).

We have previously investigated the specificity of the CD5-2 against 2 other miR-27–verified targets, semaphoring 6A (SEMA6A) and peroxisome proliferator-activated receptor gamma (PPARγ) [26]. In addition, when the 1,166 predicted targets for miR-27a were analyzed for complementarity to CD5-2, the closest match still displayed 2 out of 15 oligonucleotide mismatches and therefore is unlikely to be a target. Thus, CD5-2 shows high selectivity for VE-cadherin as the target.

RNA sequencing (RNA-seq) has been used to identify VE-cadherin–mediated downstream signaling [54]. To further confirm CD5-2 works through VE-cadherin, we applied RNA-seq–based transcriptomic analysis to control- or CD5-2–treated ECs with or without VE-cadherin expression. In ECs with VE-cadherin expression (*VE-cadherin*^*+/+*^), the results show 585 differentially expressed genes (DEGs) in CD5-2–treated ECs compared with control-treated ECs (*P* < 0.05; false discovery rate [FDR] ≤ 0.005, Fig 6Fi). The Gene Set Enrichment Analysis (GSEA) revealed CD5-2 significantly down-regulated the gene sets involved in neutrophil arrest/transmigration pathways such as extracellular matrix (ECM) receptor interaction [55], focal adhesion [56], platelet homeostasis [57], and platelet-derived growth factor (PDGF) signalling [58] (Fig 6Fii). In addition, down-regulated DEGs also included some highly expressed genes that were not uncovered by GSEA but have been reported to be involved in neutrophil arrest/transmigration, including Notch homolog 1 (*Notch1*) [59,60], endoglin (*ENG*) [61], a disintegrin and metalloproteinase with thrombospondin motifs 7 (*ADAMTS7*) [62], and leukocyte receptor cluster member 8 (LENG8) [63] (Fig 6Fiii). ECs without VE-cadherin expression (VE-cadherin null ECs: *VE-cadherin*^*−/−*^) express miR-27a (Ct value is approximately 29 when measured in 10-ng tRNA by real-time PCR). In stark contrast to expression seen in normal ECs, in *VE-cadherin*^*−/−*^ ECs, no gene was significantly differently expressed between the control- and CD5-2–treated groups when using the same cutoff criteria (S10 Fig), suggesting VE-cadherin is essential for the CD5-2–mediated gene changes [64].

## Discussion

VE-cadherin is critical in the maintenance of vascular integrity [65], and abnormal VE-cadherin expression is seen in CCMs [28,66] (Figs 1A and 2B). The work herein describes VE-cadherin as a valid therapeutic target for restoration of integrity and normalization of the vasculature in CCM development. To date, there have been limited ways of increasing the levels of an endogenous gene. We show that CD5-2, a microRNA TSB preventing miR-27a/VE-cadherin interaction, restores the levels and localization of VE-cadherin in the endothelium of CCM lesions, leading to inhibition of associated inflammation and lesion development. Our results provide an innovative therapeutic strategy for CCMs.

There are three striking observations concerning the effect of blocking miR-27a/VE-cadherin interaction by CD5-2 on CCM lesion development. Firstly, CD5-2 is able to inhibit the total volume of lesions especially the size of the large lesions in *Ccm2*^*ECKO*^ mice, indicating that CD5-2 is able to inhibit lesion growth. Secondly, CD5-2 showed strong inhibition on established large lesions and suggests CD5-2 may promote the regression of the lesion, or at least be able to stabilize established lesions and to inhibit their growth throughout the time frame of the experiment. At present, there is no technique available to give the resolution and sensitivity that would allow the analysis of the progression of individual lesions over the time period of the experiment. The fact that CD5-2 showed strong inhibition on both the number and size of the established large lesions would suggest that it, or other small molecules that lead to increased VE-cadherin expression and localization, may be therapeutically beneficial, because large lesions have more severe disruptive inter-endothelial junctions, as shown by disorganized VE-cadherin staining, indeed, in humans, these are the lesions more likely to hemorrhage [5,23]. Furthermore, lack of significant inhibition in the number of small lesions upon CD5-2 treatment suggests potentially that miR-27a/VE-cadherin may play a less important role in the initiation of CCM development than in the growth of CCM lesions. KLF2/4 that acts upstream of miR-27a, or thrombospondin 1 (TSP1), TLR4, and other unknown pathways, may initiate CCM lesion development. The lack of effect on lesion number may also be due to the treatment time frame of CD5-2. P6 is the time earliest lesions can be detected by immunohistochemistry. A treatment regime earlier than P6 may be required for CD5-2 to prevent lesion initiation.

In CCM lesion, CD5-2 treatment induces some vessels to show increased levels of VE-cadherin (Fig 2Ai) and some vessels to show improved EC junctional staining (Fig 2Aii). The difference in response to CD5-2 is not known at this stage, and we cannot relate these different responses to the size of a lesion, which is subject to the shape of a lesion and where the lesion is cut. CD5-2 also affects tight junctional molecules such as ZO-1. In normal vessels, ZO-1 is junctional (S4Bi Fig), and although CD5-2 increases ZO-1 expression levels, little junctional localization is evident (S4Bii Fig). Because tight junctions form after adherens junctions are established [67], this may suggest that the junctions are still in the process of maturing. ZO-1, together with is partner junctional adhesion molecule-A (JAM-A), acts as an essential unit for tight junction assembly and regulation of the tension between ECs and barrier integrity [68]. Thus, together with our previous studies showing increases in Tie-2/protein kinase B (Akt)/endothelial nitric oxide synthase (eNOS) signalling pathways in ECs [25], increased VE-cadherin expression and normalized localization induced by CD5-2 have profound effects to regulate the multiple pathways associated with junctional integrity.

The third noteworthy effect is the impact of CD5-2 on the inflammatory cell accumulation. Inflammation is associated with increasing vascular permeability and infiltration of immune cells, both key features of CCM lesions and both controlled by VE-cadherin [11,69,70]. On one hand, CD5-2 inhibits vascular permeability, thus limiting a key aspect of the pathology [5,10,11]. On the other hand, CD5-2 also significantly inhibited CCM depletion–induced inflammation evidenced by decreased ICAM-1 and NF-κB activation in vitro and reduced ICAM expression and numbers of CD11b^+^–expressing inflammatory cells associated with lesions. The inhibition of neutrophil transmigration was recapitulated in a 2-cell system in vitro (Fig 6E), where only the endothelium is treated with CD5-2, thus strongly supporting the effects of CD5-2 being mediated through ECs. Of note, paracellular transmigration of neutrophils requires transient junctional disassembly of EC junctions because they migrate between adjacent cells, particularly through preexisting gaps at cell borders where three ECs meet [71]. In this study, we observed that neutrophils in the control group preferentially transmigrated through the EC junctions, while CD5-2 significantly blocked this process. Interestingly, we have previously observed CD5-2 exhibits selectivity on the inflammatory infiltrate, promoting the CD8^+^ T-cell population of cells into tumors, whereas inhibiting neutrophils [25]. This is in line with the role of normalized vasculature, which benefits the treatment of tumors [72]. Whether this selectivity exerts beneficial effects on the treatment of CCMs requires further investigation.

Loss of CCM function results in enhanced KLF2/4 levels that are critical in CCM development [3]. The role of KLF2/4 in CCM is interesting given that KLF2/4, especially KLF2, is essential for proper vascular function [73,74]. This could be explained by the existence of a critical protective range of KLF2/4 levels in various vascular beds. The level of expression of KLF2/4 for each vascular bed may be linked to blood flow [48]. For example, in arterials, KLF2/4 are normally highly expressed and associated with vascular stability and lack of inflammation [74,75]. In diseases such as arteriovenous malformations (AVMs), Alk1 or ENG (two key genes for AVMs) depletion induced higher-than-normal KLF2/4 expressions, and these increased levels were non-protective [76]. In the brain capillary-venous system, where CCM lesions develop, the ECs express the lowest levels of KLF2/4 in the human body (Gene expression for KLF2 ENSG00000127528.5, KLF4 ENSG00000136826.10, GTEx Analysis Release V7, dbGaP Accession phs000424.v7.p2). Loss-of-function mutations of CCM genes result in higher than normal expression of KLF2/4 for the capillary-venous system, and this increase is associated with CCM development. We further show here that up-regulation of KLF2/4 induces miR-27a, leading to negative regulation of VE-cadherin expression and vascular integrity. Intriguingly, miR-27a is also up-regulated in the plasma from patients with hereditary hemorrhagic telangiectasia (HHT), a vascular malformation also characterized by abnormal vascular integrity and fragile lesions [22,77]. Thus, miR-27a could be a common regulator of vascular integrity, especially in the cerebral vascular system.

Collectively, our results confirm VE-cadherin as an important therapeutic target. Given that miR-27a is increased in other diseases characterized by vascular leak [27] and in an inflammatory environment [25], CD5-2 has potential to be therapeutically beneficial in a broader stage than CCM. Our data suggest that the gene regulation induced by CD5-2 requires VE-cadherin expression (S10 Fig). Considering the extensive downstream signalling linked to VE-cadherin and, because miR-27 is also present in normal ECs, disturbing the equilibrium of miR-27a and VE-cadherin expression could lead to increases of VE-cadherin in normal ECs. Although lack of VE-cadherin has been shown to be harmful and has close links with diseases [25,27,78], little evidence suggests that small levels of overexpression of VE-cadherin causes any side effect. In addition, EC junctional molecules, including VE-cadherin, are tightly regulated by many factors, including phosphorylation, dephosphorylation, and association with other proteins [70]. MiR-27a may or may not be one of these regulatory factors in homeostasis. The lack of major abnormalities in the cortical vascular density in WT animals treated with CD5-2 (S6 Fig) supports the possibility that miR-27a may not be a major regulator in the quiescent vasculature. However, a thorough toxicity study of CD5-2 or any other small molecule targeting VE-cadherin, as is required in preclinical development of a drug, would pave the way for its application in clinic.

Finally, because multiple pathways are activated with loss of CCM function, combinations with CD5-2 or alternate drugs that target VE-cadherin, together with drugs that are RhoA/ROCK [23], TSP1 [79], TLR4 [13] directed or targeting other miRNAs involved in vascular integrity, such as miR-132 [80], may exert further additive or synergistic effects.

## Materials and methods

### Ethics statement

HUVECs were isolated from donated cords with approval from the Royal Prince Alfred Hospital Human Ethics Committee (permit and approval number X16-0225). The umbilical cords were anonymous (donors nonidentifiable) and informed consent was given for their use. All animal experiments were performed under protocols approved by the New South Wales Local Sydney Health District Ethics Committees (permit and approval numbers 2013–029, 2017–002, and 2017–028). All animal procedures conform to the guidelines from National Health and Medical Research Council of Australia. All human samples from CCM patients were collected and analyzed at Sun Yat-sen University, as approved by the Sun Yat-sen University Ethical Committee (permit and approval number SYSEC-KY-KS-2018-146). This study was conducted in accordance with the Declaration of Helsinki.

### Reagents and cells

The TSBs were synthesized by Axolabs (Kulmbach, Germany). Control TSB is similar in sequence length and design to the TSB CD5-2, in terms of modifications and oligonucleotide length. However, it has no homology to any known mouse, rat, or human miRNA or mRNA sequence. The design and sequence of TSB control and CD5-2 were the same as published previously: CD5-2, 3′-ACTTCGTUGACACUT-5′; Control, 3′-TCCAGAGATGGTUGA-5′ [25,26]. Human cerebral microvascular EC line hCMEC/D3 was a gift from Dr. Georges Grau (Bosch Institute, the University of Sydney) and maintained in Endothelial Cell Basal Medium-2 (CC-3156, Lonza) supplemented by SingleQuots Kit (CC-4176, Lonza). HUVECs were isolated and cultured as previously described [25,26] and used at passage 2. The umbilical cords were anonymous (donors nonidentifiable), and informed consent was given for their use. *VE-cadherin* null ECs were maintained in DMEM (11995065, Thermo Fisher Scientific, Waltham, MA) with 10% FBS (Thermo Fisher Scientific, Waltham, MA).

Primary mouse cerebellar ECs were isolated as described previously [13]. MiniMACS separator and starting kit (130-090-312, Miltenyi Biotec, Bergisch Gladbach, Germany) were employed for the isolation. In brief, brain microvascular fragments were dissected from control, *Ccm1*^*ECKO*^, and *Ccm2*^*ECKO*^ mice (P8). Each preparation was a pool of 2–5 mice. After digestion [11], the cell pellet was passed through a sterile 70-μm Nylon mesh followed by centrifugation (5 minutes, 300*g*). Cell pellets were incubated for 15 minutes at 4°C with microbeads previously coated with anti-CD31. Finally, ECs were separated by MS column in the MACS separator.

### siRNA knockdown and drug treatments in cultured ECs

siRNAs were used for the knockdown experiments. For siRNA transfection, ECs were transfected with siRNA against CCM1(s2510), CCM2(s38037), KLF2 (s20270), KLF4 (s17793), or scrambled siRNA (4390843) with Lipofectamine RNAiMAX Reagent (13778150, all siRNAs and reagents from Thermo Fisher Scientific, Waltham, MA) and then maintained overnight. Culture medium was then changed. For co-transfection, siRNA transfection medium was maintained for 4 hours. The second transfection with TSB CD5-2 or control was then performed with HiPerFect Transfection Reagent (301705, Qiagen, Hilden, Germany) and maintained overnight as described before. Medium was changed to culture medium in the next day. Cells were collected 24 or 48 hours after transfection for permeability assay or RNA/protein assay, respectively. siRNAs used were as follows: CCM1 (4390843, Thermo Fisher Scientific, Waltham, MA), human CCM2 (s2510, Thermo Fisher Scientific, Waltham, MA), mouse Ccm2 (s103626, s103627, s103628 Thermo Fisher Scientific, Waltham, MA), and scramble (s38037, Thermo Fisher Scientific, Waltham, MA).

### RNA and microRNA expression assay

Total RNA from freshly isolated mouse brain ECs was prepared with RNeasy micro kit (74004, Qiagen, Hilden, Germany) according to the protocol. Total RNA from cultured ECs was isolated by Trizol (15596018, Thermo Fisher Scientific, Waltham, MA), as previously reported [29]. High-Capacity cDNA Reverse Transcription Kit (4368814, Thermo Fisher Scientific, Waltham, MA) was used for cDNA synthesis. Real-time qPCR was performed with TaqMan Gene Expression Master Mix (4369016, Thermo Fisher Scientific, Waltham, MA) or TaqMan Universal PCR Master Mix (4324018, Thermo Fisher Scientific, Waltham, MA) using the specific TaqMan probe (432401, Thermo Fisher Scientific, Waltham, MA). Human probes used were as follows: ACTIN (Hs01060665_g1), KRIT1/CCM1 (Hs01090981_m1), CCM2 (Hs01123855_m1), KLF2 (Hs00360439_g1), and KLF4 (Hs00358836_m1). Mouse probes used were as follows: Actin (Mm00607939_s1), Krit1/Ccm1 (Mm01316552_m1), Ccm2 (Mm00524581_m1), Klf2 (Mm00500486_g1), and Klf4 (Mm00516104_m1). microRNAs used were as follows: RNU6B (001093), RNU48 (001006), and miR-27a (000408). qPCR was performed on a Biorad real-time PCR machine. The relative RNA and microRNA amount was calculated with the 2^−ΔΔCT^ method. β-actin was used as housekeeping control for gene expression assay. RNU48 or RNU6 small nuclear RNA is used as the reference gene for microRNA expression measurement.

### Western blotting

Proteins were isolated and quantified as described before [29]. Western blotting assay were done according to the protocol from Invitrogen. In brief, equal amount of proteins were loaded on NuPAGE 4%–12% Bis-Tris Protein Gels (Thermo Fisher Scientific, Waltham, MA), and separated and transferred to PVDF membrane (polyvinyl difluoride, EMD Millipore, Burlington, MA) by using Mini Gel Tank system (Thermo Fisher Scientific, Waltham, MA). After being blocked with 5% skim milk in PBS-Tween 20 (0.05%), the PVDF membranes were incubated with primary and HRP-linked secondary antibodies. Bands were visualized using ECL or ECL Plus Western blotting Detection Reagents (GE Healthcare, Chicago, IL) on ChemiDoc MP system (Bio-rad, Hercules, CA). Densitometric analysis was performed using Fiji imageJ software (Version: 2.2.2-rc-34/1.50a, National Institutes of Health). The following antibodies were used for Western blotting: VE-cadherin goat (1:1,000, sc-6458, Santa Cruz Biotechnology, Dallas, TX), ICAM-1 rabbit (1:1,000, 4915, Cell Signaling Technology, Danvers, MA), Phospho-NF-κB p65 rabbit (Ser536) (1:1,000, 3033, Cell Signaling Technology, Danvers, MA), claudin-5 rabbit (1:1,000, sc-28670, Santa Cruz Biotechnology, Dallas, TX), α-Tubulin mouse (1:1,000, T6074, Sigma-Aldrich, ‎St. Louis, MO), and β-actin rabbit (1:1,000, A2103, Sigma-Aldrich, ‎St. Louis, MO). HRP-linked secondary antibodies: goat anti-mouse IgG HRP (1:5,000, 10004302, Cayman Chemical, Ann Arbor, MI), goat anti-rabbit IgG HRP (1:5,000, 10004301, Cayman Chemical, Ann Arbor, MI), and donkey anti-goat IgG-HRP (1:5,000, sc-2020, Santa Cruz Biotechnology, Dallas, TX).

### RhoA activity assay

Measurement of active RhoA levels was determined using the GLISA assays (Cytoskeleton inc, Denver, CO). Briefly, control, CCM1, or CCM2 siRNA–treated HUVECs were incubated for 1 hour in serum-free medium 199, followed by incubation in medium with 1% serum overnight. Cells were stimulated with TNF-α for 1 hour and lysed for the measurement of RhoA activity according to the manufacturer’s protocol.

### Transwell permeability assay in cultured ECs

For in vitro permeability assay, hCMEC/D3 cells were plated onto fibronectin-coated 3-μm-pore transwell inserts (Corning Costar, Acton, MA). After 48 hours, 40-KDa FITC-conjugated dextran (Sigma-Aldrich, ‎St. Louis, MO) was added to the upper chamber, and medium was taken from the lower chamber (20 μL) 20 minutes later. The amount of fluorescence was measured using a POLARstar Omega microplate reader (BMG Labtech, Ortenberg, Germany).

### Induction of the neonatal CCM mouse model

*Ccm1*^*fl/+*^*;Tg(Tie2-Cre)*, *Cdh5-CreErt2*, *Ccm1*^*fl/fl*^, and *Ccm2*^*fl/fl*^ animals have been previously described [8,12,13]. *Cdh5-CreErt2;Ccm1*^*fl/+*^ or *Cdh5-CreErt2;Ccm2*^*fl/+*^ were crossed with *Ccm1*^*fl/fl*^ or *Ccm2*^*fl/fl*^ to generate *Cdh5-CreErt2;Ccm1*^*fl/fl*^ (*Ccm1*^*ECKO*^) or *Cdh5-CreErt2;Ccm2*^*fl/fl*^ (*Ccm2*^*ECKO*^). To induce *Ccm2* gene EC-specific deletion in neonatal mice, at P1, 40 μg of 4-hydroxytamoxifen (4OHT, Sigma-Aldrich, ‎St. Louis, MO) dissolved in ethanol and corn oil was injected intragastrically to pups.

### Permeability in CCM neonatal animal

Permeability was measured as described previously with modification [11]. In brief, animals (P6) first received Ctrl or CD5-2 treatment. After 2 days (P8), they received IP injection of Alexa Fluor 555 cadaverine (Thermo Fisher Scientific, Waltham, MA) (25 μg per 1 g). Two hours later, pups were euthanized by inhalation of CO_2_ and then underwent intracardiac perfusion with PBS. Brains were dissected immediately and fixed in 4% PFA at 4°C overnight. Fluorescence in the hindbrain was measured by M205FA stereomicroscope (Leica Biosystems, Wetzlar, Germany) with a fluorescent lamp and filters for Cy3 (555/60 Ex and 610/75 Em). Intensity of fluorescence was quantified by Fiji imageJ software (version 2.2.2-rc-34/1.50a, National Institutes of Health).

### Effect of CD5-2 on retinal vascular development

The retina vasculature was stained as described before [81]. In brief, retinas were equilibrated with PBLEC (1 mM MgCl_2_, 1 mM CaCl_2_, 0.1 mM MnCl_2_, and 1% Triton X-100 in PBS) and incubated with biotinylated isolectin-B4 (1:25, Vector Laboratories, Burlingame, CA) overnight, washed, then incubated with streptavidin Alexa Fluor-594 (1:500, Thermo Fisher Scientific, Waltham, MA) and mounted with ProLong Gold anti-fade reagent (Thermo Fisher Scientific, Waltham, MA). Images were acquired using a Leica SP5 confocal microscope (Leica Biosystems, Wetzlar, Germany). Lesions were marked as the areas of condensed vascular plexus at the leading edge of the retinal vasculature. Fiji ImageJ software was used to quantify vascular coverage of the whole retina and lesions.

### VE-cadherin immunostaining in human CCM lesions

Surgical samples from human patients with CCM lesions were resected at Sun Yat-sen University. Normal-sized vessels (pseudonormal) present in samples from CCM patients were used as internal control. Six patients (3 male, 3 female) between 25 and 52 years old were included in this study. Tissue sections were paraffin-embedded and stained with rabbit anti–VE-cadherin (1:1,000, ab33168, Abcam,Cambridge, United Kingdom).

### IP TSB injection and organ dissection

TSB control and CD5-2 were administered to *Ccm2*^*ECKO*^ neonatal CCM disease model. At P6 and P8 (early stage treatment) or P12 and P14 (late stage treatment), a 30 mg/kg dose of TSB at 7.5 mg/mL dissolved in sterile nuclease free H_2_O was IP injected to *Ccm2*^*ECKO*^ mice and WT littermates. Pups were euthanized at P12 or P19 by inhalation of CO_2_ and then underwent intracardiac perfusion with 2% paraformaldehyde (PFA, Sigma-Aldrich, ‎St. Louis, MO) in 0.1 M PBS. Brains and eyes were dissected immediately and fixed in 4% PFA at 4°C for downstream measurement.

### Miles assay for assessment of dermal vascular leakage

Permeability in the skin was measured as previously reported [26,29]. In brief, the TSB inhibitors or control were injected intradermally at 6 sites on the back of 8–20-week-old *Ccm1*^*fl/+*^*;Tg(Tie2-Cre)* mice and WT littermates. After 24 hours, 200 μL of 0.5% Evans blue dye was injected intravenously to animals. Thirty minutes later, PBS alone or PBS containing 100 ng of VEGF-165 (R&D Systems, Minneapolis, MN) was injected intradermally into the same site as the TSB inhibitors. After another 30 minutes, mice were euthanized by inhalation of CO_2_. Biopsies of the injected area were taken and Evans blue eluted from biopsies by Formamide (Sigma-Aldrich, ‎St. Louis, MO) overnight at 56°C. Absorbance of Evans blue was measured at 620 nm using a POLARstar Omega microplate reader (BMG Labtech, Ortenberg, Germany).

### In situ hybridization

Detection of CD5-2 in blood vessels was done as previously described [25]. In brief, animals (P6) were treated with CD5-2 via IP injection. After 6 hours, they underwent intracardiac perfusion with PBS. Hindbrains were then dissected and kept in 20% sucrose overnight. The hindbrain was cut into 10-μm sections. The probe for hybridization was target specific for CD5-2 (5′-FAM-IT mG mA mA IG mC mA mA IC mU mG IT mG mA IA-3′). Hybridization temperature cycle and fluorescence detection was the same as the one used previously [25]. After washes and subsequent staining with anti-CD31, we found the normal dilutions of 1:200 required for routine CD31 staining did not work. CD31 staining was weakly detectable even after we used a dilution of 1:50, thus suggesting that there has been a decrease in signal, likely because of the time and manner of sample preparation. Alternatively, the identification of vasculature in the brain sections was aided by the autofluorescence or native fluorescence of elastin fibers within the vasculature. This is widely reported across different tissues and can be detected at wavelengths similar to FITC [82, 83]. Using the native fluorescence as a guide, we could pinpoint the localization of CD5-2 to the brain vasculature.

### Histology and immunofluorescence

Dissected hindbrains were fixed in 4% PFA at 4°C for 2–3 hours, then incubated in 30% sucrose (Sigma-Aldrich, ‎St. Louis, MO) for overnight. Tissues were then frozen in Tissue-Tek O.C.T. Compound (Sakura Finetek, Torrance, CA) and after freezing, 10-μm sections of hindbrain tissue were cut onto Superfrost Plus slides (Thermo Fisher Scientific, Waltham, MA). The sections from littermate control and experimental animal sections were placed on the same slide and immunostained at the same time under identical conditions using our standard protocol [25]. For primary staining, the following antibodies were used for immunostaining: rat CD31 (1:200, 553370, BD Biosciences, San Jose, CA), rabbit ICAM-1 (1:200, ab124759, Abcam,Cambridge, United Kingdom), rabbit pMLC2 (1:200, 3674S, Cell Signaling Technology, Danvers, MA), rat CD144 (1:200, 555289, BD Biosciences, San Jose, CA), goat VE-cadherin (1:1,000, sc-6458, Santa Cruz Biotechnology, Dallas, TX), and rat CD11b (1:200, 53-0112-82, eBiosciences, San Diego, CA). Secondary antibodies were as follows: anti-rabbit Alexa Fluor 594, anti-Donkey Alexa Fluor 488, anti-rat Alexa Fluor 488, or anti-rat Alexa Fluor 647 antibodies (1:600; Thermo Fisher Scientific, Waltham, MA) in PBS. Cell nuclei were stained with DAPI. Images were taken by SP5 confocal microscope (Leica Biosystems, Wetzlar, Germany) using the same parameters and were subsequently overlaid using Fiji imageJ software (version 2.2.2-rc-34/1.50a, National Institutes of Health). To quantify VE-cadherin, cells immunostained for VE-cadherin were converted to black and white based on the same threshold between TSB control- and CD5-2–treated mice, and VE-cadherin expression intensity was defined as the mean number of pixels above the threshold in the area lining the CCM lesions.

### SEM

The structures of endothelium of CCM animal were analyzed by SEM as previously reported, with modification [84]. In brief, animals were treated with TSB control or CD5-2 at P6 and P8. At P12, animals were euthanized by inhalation of CO_2_, followed by intracardiac perfusion with fixative (pH 7.4, 15 mL per animal) containing 0.25% (w/v) glutaraldehyde (ProSciTech, Queensland, Australia) EM grade, 2% paraformaldhyde (Sigma-Aldrich, ‎St. Louis, MO), 20 mmol/L calcium chloride (Ajax Chemicals, New South Wales, Australia), 2% (w/v) sucrose (Sigma-Aldrich, ‎St. Louis, MO), and 0.1 mol/L cacodylate (ProSciTech, Queensland, Australia). Hindbrains were then dissected and kept in fixative overnight before being analyzed by a JEOL 6340 SEM, as previously described [84] (SEMTech Solutions, MA).

### X-ray microCT-based quantification of CCM lesions

X-ray microCTs were employed to quantify CCM lesions, as previously reported [8]. In brief, after fixation in 4% formaldehyde, hindbrains were then detached from mid/forebrains and soaked for 48 hours in 2% osmium tetroxide solution (Sigma-Aldrich, ‎St. Louis, MO) and washed several times with water before being scanned by using Xradia MicroCT system (Xradia MicroXCT-400, ZEISS, Oberkochen, Germany). The raw images were continuously scanned 2D stacks. Images where then processed by 3D image processing software Avizo (version 9.3.0, FEI Visualization Sciences Group, Hillsboro, OR). Lesions were identified by their lower grayscale intensity and the shape of the feature, together with the “Magic Wand” function from Avizo, which is able to localize the labelled individual lesions across multiple cross sections. These stacks were then reconstructed to 3D by using Avizo. Finally, hindbrain image was overlaid with the labeled lesions in Avizo to attain the desired spatially significant view. The volume of hindbrain and individual lesions were automatically measured with the software.

### Neutrophil adhesion assay

The live-cell microscopy flow model has been described [85]. In brief, human polymorphonuclear neutrophils (PMNs) were isolated from blood by 20% dextran (1:1) sedimentation at room temperature for 40 minutes. HUVECs treated with control and CD5-2 were plated onto the μ-Slide I Luer (Ibidi, Planegg, Germany) to grow to confluence. EC monolayers were then stimulated with 5 ng/mL TNF-α (R&D system, Minneapolis, MN) for 4 hours prior to shear stress treatment. The neutrophils in HUVEC medium (10^6^ cells/mL) were added into the ibidi pump system (Ibidi, Planegg, Germany). The dynamic adhesion of neutrophils onto control or CD5-2–treated HUVECs was recorded after 6 minutes of rolling (2 dyn/cm^2^ constant shear stress) using an inverted phase-contrast microscope (Nikon Eclipse TE2000, Nikon,Tokyo, Japan) connected to a Nikon Digital Sight camera. Neutrophils per field were counted by Fiji ImageJ software (version 2.2.2-rc-34/1.50a, National Institutes of Health).

### Live cell fluorescent imaging of neutrophil transmigration

The live-cell fluorescent imaging of neutrophil transmigration was recorded using laser scanning confocal microscopy (Leica TCS SP5, Leica Biosystems, Wetzlar, Germany). In brief, HUVECs treated with control or CD5-2 for 48 hours were plated into the ibidi Culture-Insert 2 Well in the 35-mm petri dish (MatTek, Emu Plains NSW 2750) to form the EC monolayer. The isolated PMN labeled with CellTracker green dye (Thermo Fisher Scientific, Waltham, MA) were added onto the top of TNF-α–stimulated HUVECs, which were stained with VE-cadherin antibody (Cell Signaling Technology, Danvers, MA). The transmigration processes of PMN across the endothelium treated with control and CD5-2 were recorded by time-lapse confocal microscopy for 1 hour.

### RNA-seq and GSEA

The RNA-seq was done as previously described [85]. Total RNAs were extracted from HUVEC or VE-cadherin null ECs treated with control or CD5-2 using RNeasy Plus Mini Kit (Qiagen, Hilden, Germany). A total of 3–5 biological replicates were pooled for RNA-seq. Libraries were prepared using the NEBNext UltraTM RNA Library Prep Kit for Illumina (San Diego, CA). After library construction, libraries were diluted to 1.5 ng/uL with the preliminary quantitative result by Qubit2.0. Library fragments were purified with AMPure XP system (Beckman Coulter, Brea, CA) and assessed by the Agilent Bioanalyzer 2100 system to detect the insert size. Library preparations were sequenced on an Illumina Hiseq 4000 platform and 150-bp paired-end reads were generated. Reference genome and gene model annotation files were downloaded from the genome website browser (NCBI/UCSC/Ensembl) directly. Indexes of the reference genome were built using Bowtie (v2.2.3), and paired-end clean reads were aligned to the reference genome using (v2.0.12). We then used HTSeq (v0.6.1) to count the read numbers mapped of each gene and used fragments per kilo bases per million reads (FPKM) to estimate gene expression level based on the length of the gene and reads count mapped to that gene. Prior to differential gene expression analysis, for each sequenced library, the read counts were adjusted by using a weighted trimmed mean of the log expression ratios (trimmed mean of M values) [86]. DEGs were then determined using DESeq R package (v1.12.0). The resulting *P* values were adjusted using Benjamini and Hochberg’s approach for controlling the FDR. Genes with an adjusted *P* value < 0.05 and FDR < 0.005 were assigned as differentially expressed. Modified GSEA was used to assess functional significance at the level of sets of genes, as previously described [87]. Heatmaps were generated using the Multiple Array Viewer MeV_4_8 (version 10.2).

### Statistics

All mice were randomized before the treatment. Results of scanning electron microscopy were assessed blindly. Hindbrain samples were scanned by microCT blindly. All other experiments were not assessed blindly. Sample size determination was based on previous experience with similar studies. The researchers were not blinded to the distribution of treatment groups when performing experiments and data assessment. Data are expressed as mean ± SEM for the indicated number of observations. *P* values were calculated as indicated in figure legends using an unpaired, two-tailed Student *t* test or one-way ANOVA with Tukey correction for multiple comparisons. *P* < 0.05 was considered significant.

## Supporting information

S1 Fig CCM-deficient mice show abnormality in retina and brain vasculature. (A) Mouse retinas after dissection (i) and (ii) isolectin-B4 (red) and VE-cadherin (green) staining. Bar, upper panel, 200 μm; lower panel, 100 μm. (B) CCM malformations show blood-filled caverns in the hindbrain of P12 *Ccm2*^*ECKO*^ (*iECre*, *Ccm2*^*fl/fl*^) mice upon dissection. (ii) Staining of VE-cadherin in the CCM lesions of different sizes. Bar, (i) 1 mm; (ii) 8 μm. For the raw data used for quantification, see S1 Fig in S1 Data. CCM, cerebral cavernous malformation; V, vein; VE-cadherin, vascular endothelial cadherin
(TIF)

S2 Fig Loss of CCM1 or CCM2 disrupts EC junctions. (A) Real-time PCR analysis of CCM1 or CCM2 expression in HUVECs and hCMEC/D3 treated with siRNA targeting scramble (si-Ctrl), CCM1 (si-CCM1), or CCM2 (si-CCM2) (*n* = 2–4). (B) VE-cadherin and claudin-5 protein expression, analyzed by western blot, in hCMEC/D3 treated with siRNA to Ctrl, CCM1, or CCM2 (*n* = 2–3). (C) Immunostaining for VE-cadherin in HUVECs treated with control, CCM1, or CCM2 siRNAs. Representative images are shown. Bar, upper panel 100 μm; lower panel 50 μm. (D) miR-27a expression in brain ECs isolated at P5 from the WT (*n* = 2) and *Ccm2*^*ECKO*^ (*n* = 3) mice. For the raw data used for quantification, see S2 Fig in S1 Data. CCM, cerebral cavernous malformation; EC, endothelial cell; hCMEC/D3, human cerebral microvascular endothelial cells/D3; HUVEC, human umbilical vein endothelial cell; miR-27a, microRNA-27a; siRNA, small interfering RNA; VE-cadherin, vascular endothelial cadherin; WT, wild-type
(TIF)

S3 Fig CD5-2, a TSB to miR-27a/VE-cadherin, rescues key junction molecule expression. (A) Sequence of TSB control (Ctrl), CD5-2, miR-27a, and binding site on VE-cadherin 3′ UTR. TSBs were modified oligonucleotides. (B) Western blot analysis of VE-cadherin and claudin-5 in hCMEC/D3 treated with 10 nM siRNAs for scramble control (Ctrl), CCM1, or CCM2 for 4 hours, followed by transfection of 15 nM CD5-2 or controls, then cultured overnight. Molecular weights in kilodaltons are shown. Representative blots are shown (*n* = 3–5), with α-tubulin used as loading control. For the raw data used for quantification, see S3 Fig in S1 Data. S3B Fig in S1 Raw images. CCM, cerebral cavernous malformation; hCMEC/D3, human cerebral microvascular endothelial cells/D3; miR-27a, microRNA-27a; siRNA, small interfering RNA; TSB, target site blocker; VE-cadherin, vascular endothelial cadherin
(TIF)

S4 Fig CD5-2 rescues endothelial junction and inhibits CCM lesions in the animal with CCM diseases. (A) Experimental protocol for early stage treatment was as follows: Ctrl or CD5-2 (30 mg/kg) was administered by IP injection at P6 and P8, and brains and retinas were dissected at P12. (B) (i) ZO-1 staining in normal brain vessels from WT mice. (ii) CD5-2 increased ZO-1 expression in CCM lesions. Bar, 60 μm. (C) Representative HE staining of cerebellar sections from *Ccm2*^*ECKO*^ mice after treatment with Ctrl or CD5-2. Bar, 100 μm. (D) Representative image of CD31 staining in cerebellar sections from *Ccm2*^*ECKO*^ mice after treatment with Ctrl or CD5-2. Bar, 60 μm. CCM, cerebral cavernous malformation; Ctrl, control; HE, hematoxylin–eosin; IP, intraperitoneal; siRNA, small interfering RNA; WT, wild-type; ZO-1, zonula occludens-1
(TIF)

S5 Fig Late treatment of CD5-2 does not affect CCM lesion numbers. (A) Experimental protocol for late stage treatment: Ctrl or CD5-2 (30 mg/kg) was administered by IP injection at P12 and P14, and brains and retinas were dissected at P19. (B) Number of lesions from mice treated in (A) (*n* = 3, from 3 litters). Values are shown as mean ± SEM. N.S, not significant, determined by Student *t* test. For the raw data used for quantification, see S5 Fig in S1 Data. CCM, cerebral cavernous malformation; Ctrl, control; IP, intraperitoneal
(TIF)

S6 Fig Treatment of CD5-2 has no effect on development and vascularization of hindbrain and the surface of the retina. (A) Representative images of hindbrain, retina, and their vasculature from Ctrl- and CD5-2–treated WT mice. Bars, brain, 1 mm; retina, 200 μm; bottom panel left, 100 μm; right, 200 μm. Samples were dissected at P12. (B) Treatment of CD5-2 has no effect on size of hindbrain from mice treated at P6 and P8; *n* = 7 mice for control-treated group and *n* = 9 mice for CD5-2–treated group. Mice are from 4 different litters. (C) Treatment of CD5-2 has no effect on size of hindbrain from mice treated at P12 and P14 compared with Ctrl-treated mice (*n* = 4, from 4 litters). Values are shown as mean ± SEM. N.S, not significant, determined by Student *t* test. For the raw data used for quantification, see S6 Fig in S1 Data. CCM, cerebral cavernous malformation; Ctrl, control; WT, wild-type
(TIF)

S7 Fig CD5-2 rescues defect in dermal integrity in *Ccm1* heterozygous mice. (A) Miles assay of dermal permeability in heterozygous Ccm1 (*Tie2-Cre*, *Ccm1* ^*fl/+*^) mice in response to PBS or VEGF after pretreatment with Ctrl or CD5-2. (B) Quantification of Evans blue extravasation, three mice for each group. Bar, 500 μm. Values are shown as mean ± SEM. *P < 0.05, determined by one-way ANOVA with Tukey correction. For the raw data used for quantification, see S7 Fig in S1 Data. CCM, cerebral cavernous malformation; Ctrl, control; VEGF, vascular endothelial growth factor.
(TIF)

S8 Fig CD5-2 inhibits loss-of-CCM induced RhoA-ROCK activity but has no effect on KLF2/4 expression. HUVECs were treated with 10 nM siRNAs for scramble control, CCM1, or CCM2 (si-Ctrl, si-CCM1, or si-CCM2) for 4 hours, followed by transfection of 15 nM CD5-2 or Ctrl, then cultured for 48 hours. (A) RhoA activity was measured using RhoA G-LISA activation assay kit (*n* = 4). (B) Immunostaining was performed to measure pMLC. Representative images are shown. Intensity of staining was quantified from 3–5 images per treatment, *n* = 3 experiments. Bar, 60 μm. Quantification of immunoblots was by Fiji ImageJ software (*n* = 3–5). (C) mRNA levels of KLF2 and KLF4 following Ctrl or CD5-2 treatment in CCM1- or CCM2-depleted ECs, analyzed by real-time PCR analysis (*n* = 2). Data represent mean ± SEM. *P < 0.05, **P < 0.01, ****P < 0.001, determined by one-way ANOVA with Tukey correction. For the raw data used for quantification, see S8 Fig in S1 Data. CCM, cerebral cavernous malformation; Ctrl, control; EC, endothelial cell; G-LISA, G protein linked immunosorbent assay; HUVEC, human umbilical vein endothelial cell; KLF2/4, kruppel-like factor 2/4; pMLC, phospho-myosin light chain; RhoA, ras homologue A; ROCK, Rho-associated protein kinase; siRNA, small interfering RNA
(TIF)

S9 Fig CD5-2 rescues inflammatory response. (A) CD11b^+^ (yellow) leukocytes accumulate in CCM lesions treated with CD5-2. Blood vessels are stained with CD31 (red), and cell nuclei are stained with DAPI (blue). Images in all panels are representative of at least 3 mice in each group. Quantification of number of CD11b^+^ cells in CCM lesion. (B) Quantification of number of CD11b^+^Gr1^hi^ cells in MC38 tumor model (*n* = 8). (C) Quantification of percentage of transmigrated neutrophils (%) across the control- and CD5-2–treated ECs in the transwell migration assay (*n* = 3). For the raw data used for quantification, see S9 Fig in S1 Data. CCM, cerebral cavernous malformation; EC, endothelial cell
(TIF)

S10 Fig CD5-2 has no effect on gene expression profile in VE-cadherin null ECs. Heatmap of all genes between Ctrl- and CD5-2–treated VE-cadherin null ECs (*VE-cadherin*^*−/−*^). Ctrl, control; EC, endothelial cell; HUVEC, human umbilical vein endothelial cell; VE-cadherin, vascular endothelial cadherin
(TIF)

S1 Movie CD5-2 inhibits neutrophils adhesion on EC monolayer. Dynamic adhesion of neutrophils onto TNF-α–stimulated EC monolayer treated with control and CD5-2. HUVECs treated with control and CD5-2 were plated onto the μ-Slide I Luer (Ibidi, Germany). The neutrophils in HUVEC medium were added into the ibidi pump system (Ibidi, Germany). The dynamic adhesion of neutrophils onto control- or CD5-2–treated HUVECs was recorded under an inverted phase-contrast microscope after 6 minutes of rolling. EC, endothelial cell; HUVEC, human umbilical vein endothelial cell; TNF-α, tumor necrosis factor-α
(PPTX)

S2 Movie CD5-2 inhibits neutrophil transendothelial migration. Paracellular transmigration of GFP-labeled neutrophils (green) through TNF-α–stimulated endothelium (VE-Cadherin, red) in the presence of control and CD5-2 in vitro. The majority of neutrophils treated with control transmigrated via the paracellular route, as assessed in real time, by contact and passage through the area bonded by VE-cadherin (red), whereas CD5-2 inhibited neutrophil transmigration. GFP, green fluorescent protein; TNF-α, tumor necrosis factor α; VE-cadherin, vascular endothelial cadherin
(PPTX)

S1 Data Quantitation for all main and supporting figures: Figs 1, 2, 3, 4, 5, 6, S2, S3, S5, S6, S7, S8, S9 and S10. Additional information about a particular panel in a figure is included.
(XLSX)

S1 Raw images Original immunoblot gel images for Figs 6 and S3. (PPTX)

## Abbreviations

ADAMTS7: a disintegrin and metalloproteinase with thrombospondin motifs 7
Akt: protein kinase B
AVM: arteriovenous malformation
CCM: cerebral cavernous malformation
CCM1/2/3: cerebral cavernous malformations 1/2/3
CNS: central nervous system
DEG: differentially expressed gene
EC: endothelial cell
ECM: extracellular matrix
ENG: endoglin
eNOS: endothelial nitric oxide synthase
FDR: false discovery rate
FITC: fluorescein isothiocyanate
FPKM: fragments per kilo bases per million reads
GFP: green fluorescent protein
Gr: granulocyte
GSEA: Gene Set Enrichment Analysis
HE: hematoxylin–eosin
HHT: hereditary hemorrhagic telangiectasia
HUVEC: human umbilical vein endothelial cell
ICAM-1: intercellular adhesion molecule 1
IP: intraperitoneal
JAM-A: junctional adhesion molecule-A
KLF2/4: kruppel-like factor 2/4
KRIT1: krev interaction trapped 1
LENG8: leukocyte receptor cluster member 8
microCT: micro–computed tomography
miR-27a: microRNA-27a
Notch1: Notch homolog 1
P: day(s) post birth
PDCD10: programmed cell death 10
PDGF: platelet-derived growth factor
PD1: programmed cell death protein 1
pMLC: phospho myosin light chain
PMN: polymorphonuclear neutrophil
PPARγ: peroxisome proliferator-activated receptor gamma
RNA-seq: RNA sequencing
SEM: scanning electron microscope
SEMA6A: semaphoring 6A
siRNA: small interfering RNA
TGF-β: transforming growth factor-β
TLR4: toll-like receptor 4
TNF-α: tumor necrosis factor-α
TSB: target site blocker
TSP1: thrombospondin 1
VE-cadherin: vascular endothelial cadherin
VEGF: vascular endothelial growth factor
WT: wild-type
ZO-1: zonula occludens-1
