# Supplementary figures and images for: Targeting miR-27a/VE-cadherin interactions rescues cerebral cavernous malformations in mice
Source: PLoS Biol. 2020 Jun 5;18(6):e3000734. doi: 10.1371/journal.pbio.3000734 (PMC7299406; doi:10.1371/journal.pbio.3000734)

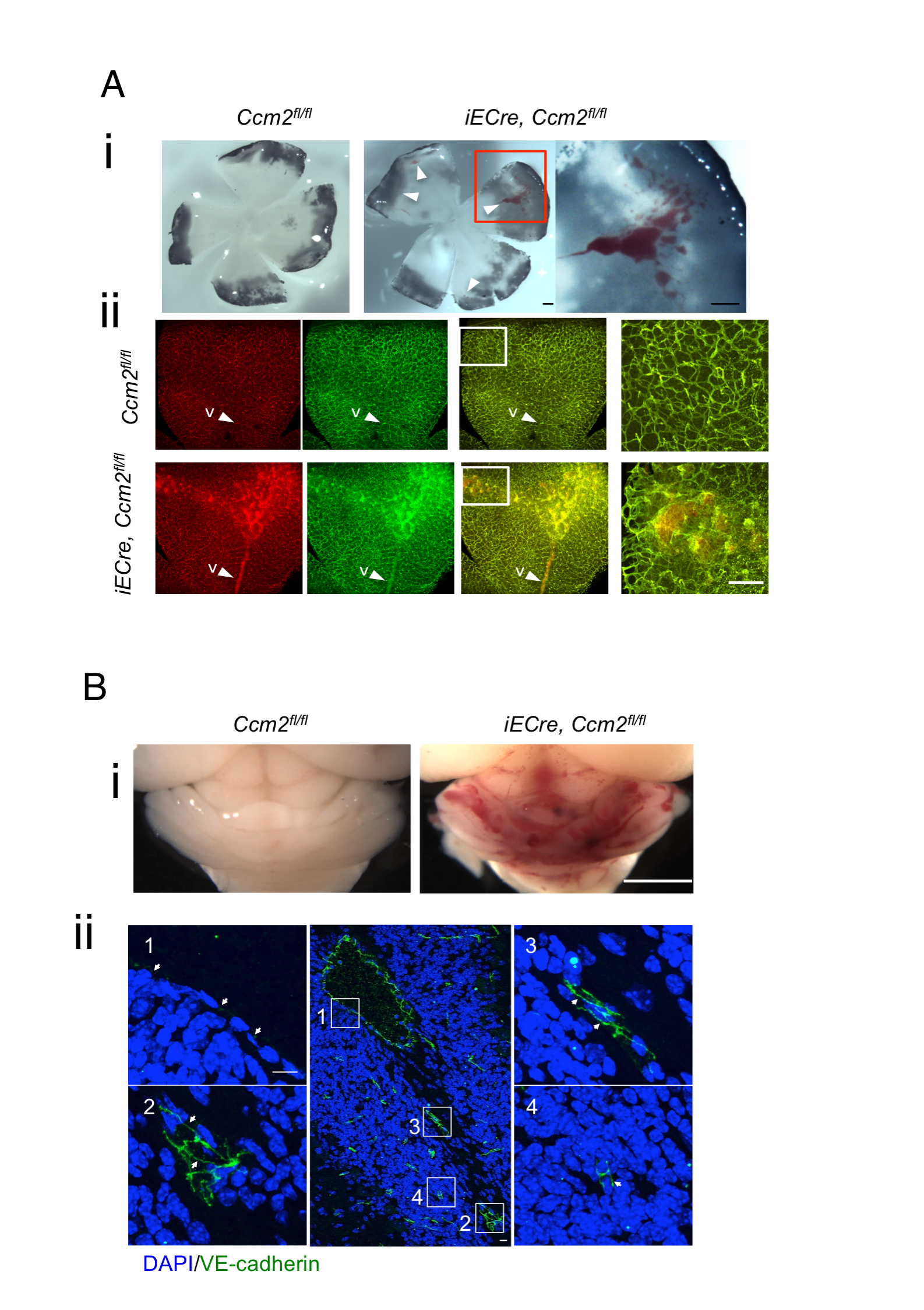

Supplement: S1 Fig — (A) Mouse retinas after dissection (i) and (ii) isolectin-B4 (red) and VE-cadherin (green) staining. Bar, upper panel, 200 μm; lower panel, 100 μm. (B) CCM malformations show blood-filled caverns in the hindbrain of P12 Ccm2ECKO (iECre, Ccm2fl/fl) mice upon dissection. (ii) Staining of VE-cadherin in the CCM lesions of different sizes. Bar, (i) 1 mm; (ii) 8 μm. For the raw data used for quantification, see S1 Fig in S1 Data. CCM, cerebral cavernous malformation; V, vein; VE-cadherin, vascular endothelial cadherin (TIF) [file pbio.3000734.s001.tif]

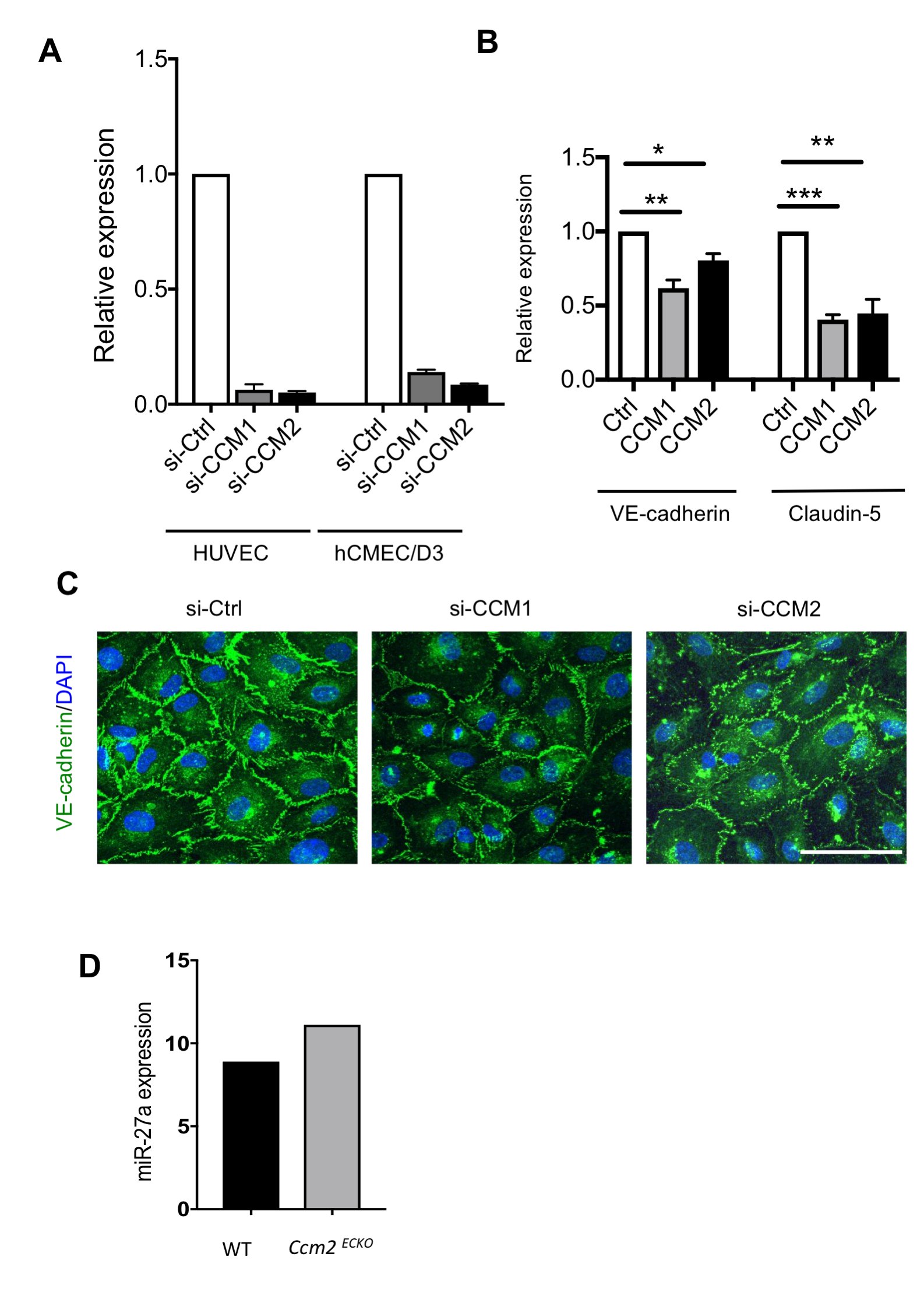

Supplement: S2 Fig — (A) Real-time PCR analysis of CCM1 or CCM2 expression in HUVECs and hCMEC/D3 treated with siRNA targeting scramble (si-Ctrl), CCM1 (si-CCM1), or CCM2 (si-CCM2) (n = 2–4). (B) VE-cadherin and claudin-5 protein expression, analyzed by western blot, in hCMEC/D3 treated with siRNA to Ctrl, CCM1, or CCM2 (n = 2–3). (C) Immunostaining for VE-cadherin in HUVECs treated with control, CCM1, or CCM2 siRNAs. Representative images are shown. Bar, upper panel 100 μm; lower panel 50 μm. (D) miR-27a expression in brain ECs isolated at P5 from the WT (n = 2) and Ccm2ECKO (n = 3) mice. For the raw data used for quantification, see S2 Fig in S1 Data. CCM, cerebral cavernous malformation; EC, endothelial cell; hCMEC/D3, human cerebral microvascular endothelial cells/D3; HUVEC, human umbilical vein endothelial cell; miR-27a, microRNA-27a; siRNA, small interfering RNA; VE-cadherin, vascular endothelial cadherin; WT, wild-type (TIF) [file pbio.3000734.s002.tif]

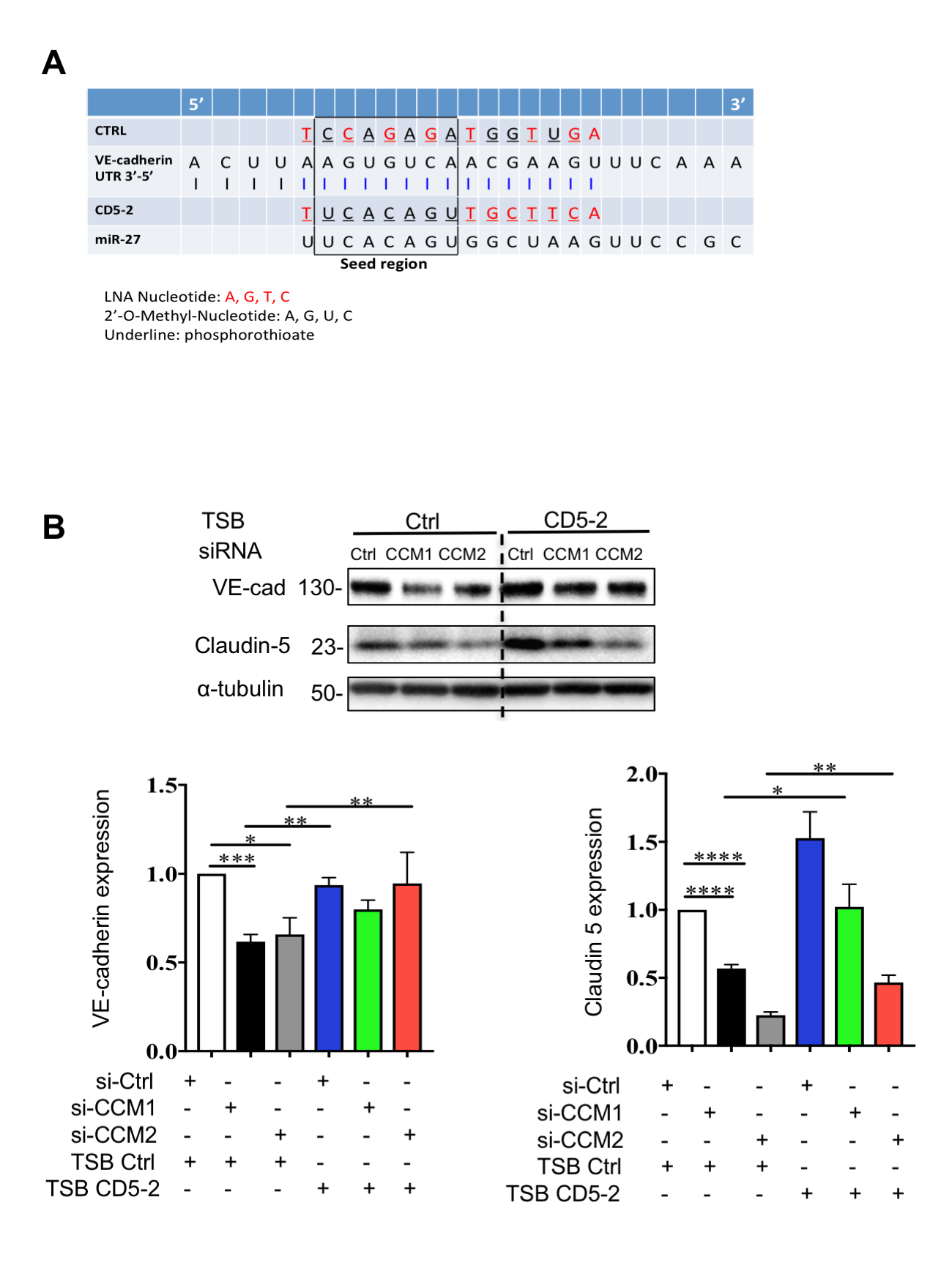

Supplement: S3 Fig — (A) Sequence of TSB control (Ctrl), CD5-2, miR-27a, and binding site on VE-cadherin 3′ UTR. TSBs were modified oligonucleotides. (B) Western blot analysis of VE-cadherin and claudin-5 in hCMEC/D3 treated with 10 nM siRNAs for scramble control (Ctrl), CCM1, or CCM2 for 4 hours, followed by transfection of 15 nM CD5-2 or controls, then cultured overnight. Molecular weights in kilodaltons are shown. Representative blots are shown (n = 3–5), with α-tubulin used as loading control. For the raw data used for quantification, see S3 Fig in S1 Data. S3B Fig in S1 Raw images. CCM, cerebral cavernous malformation; hCMEC/D3, human cerebral microvascular endothelial cells/D3; miR-27a, microRNA-27a; siRNA, small interfering RNA; TSB, target site blocker; VE-cadherin, vascular endothelial cadherin (TIF) [file pbio.3000734.s003.tif]

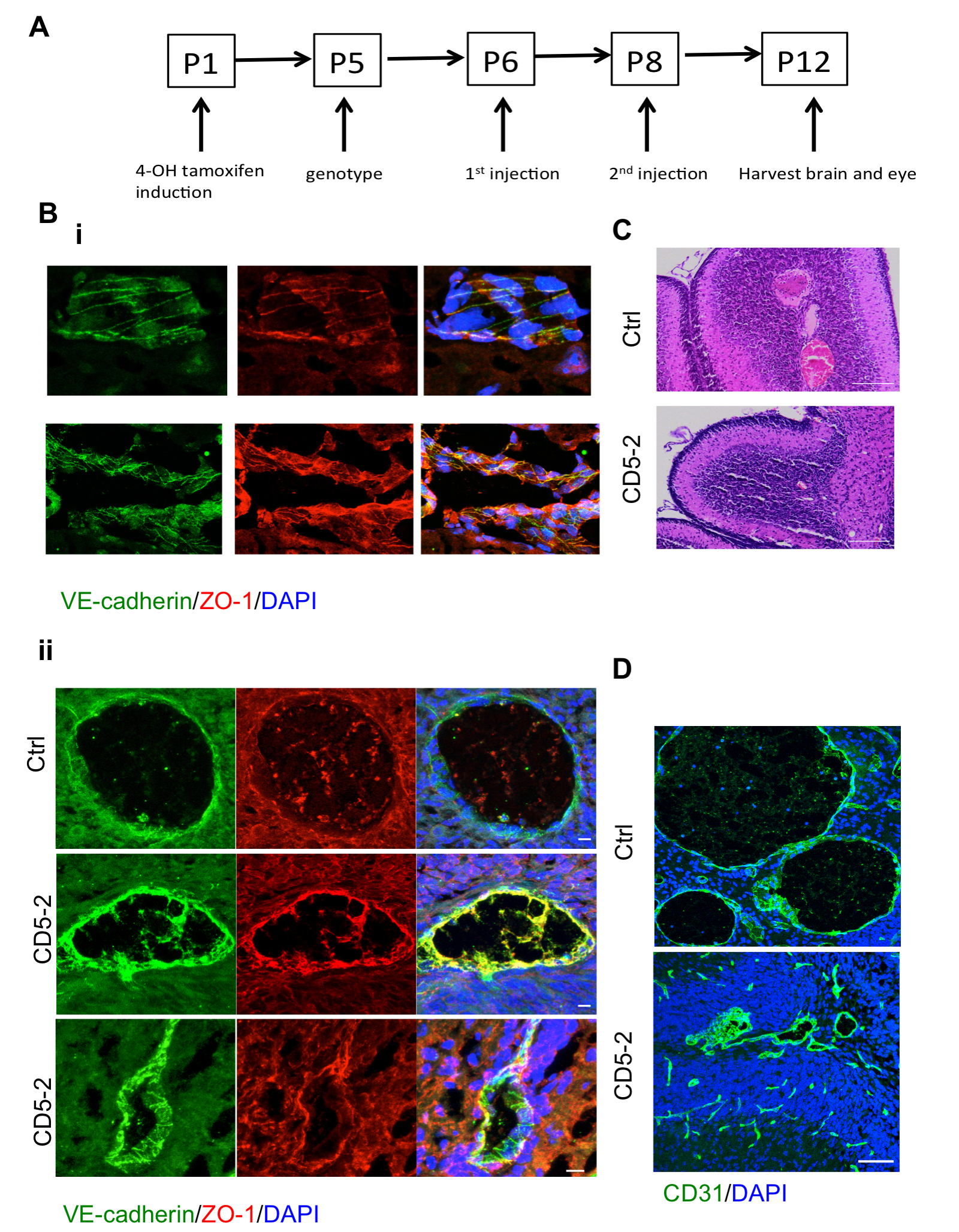

Supplement: S4 Fig — (A) Experimental protocol for early stage treatment was as follows: Ctrl or CD5-2 (30 mg/kg) was administered by IP injection at P6 and P8, and brains and retinas were dissected at P12. (B) (i) ZO-1 staining in normal brain vessels from WT mice. (ii) CD5-2 increased ZO-1 expression in CCM lesions. Bar, 60 μm. (C) Representative HE staining of cerebellar sections from Ccm2ECKO mice after treatment with Ctrl or CD5-2. Bar, 100 μm. (D) Representative image of CD31 staining in cerebellar sections from Ccm2ECKO mice after treatment with Ctrl or CD5-2. Bar, 60 μm. CCM, cerebral cavernous malformation; Ctrl, control; HE, hematoxylin–eosin; IP, intraperitoneal; siRNA, small interfering RNA; WT, wild-type; ZO-1, zonula occludens-1 (TIF) [file pbio.3000734.s004.tif]

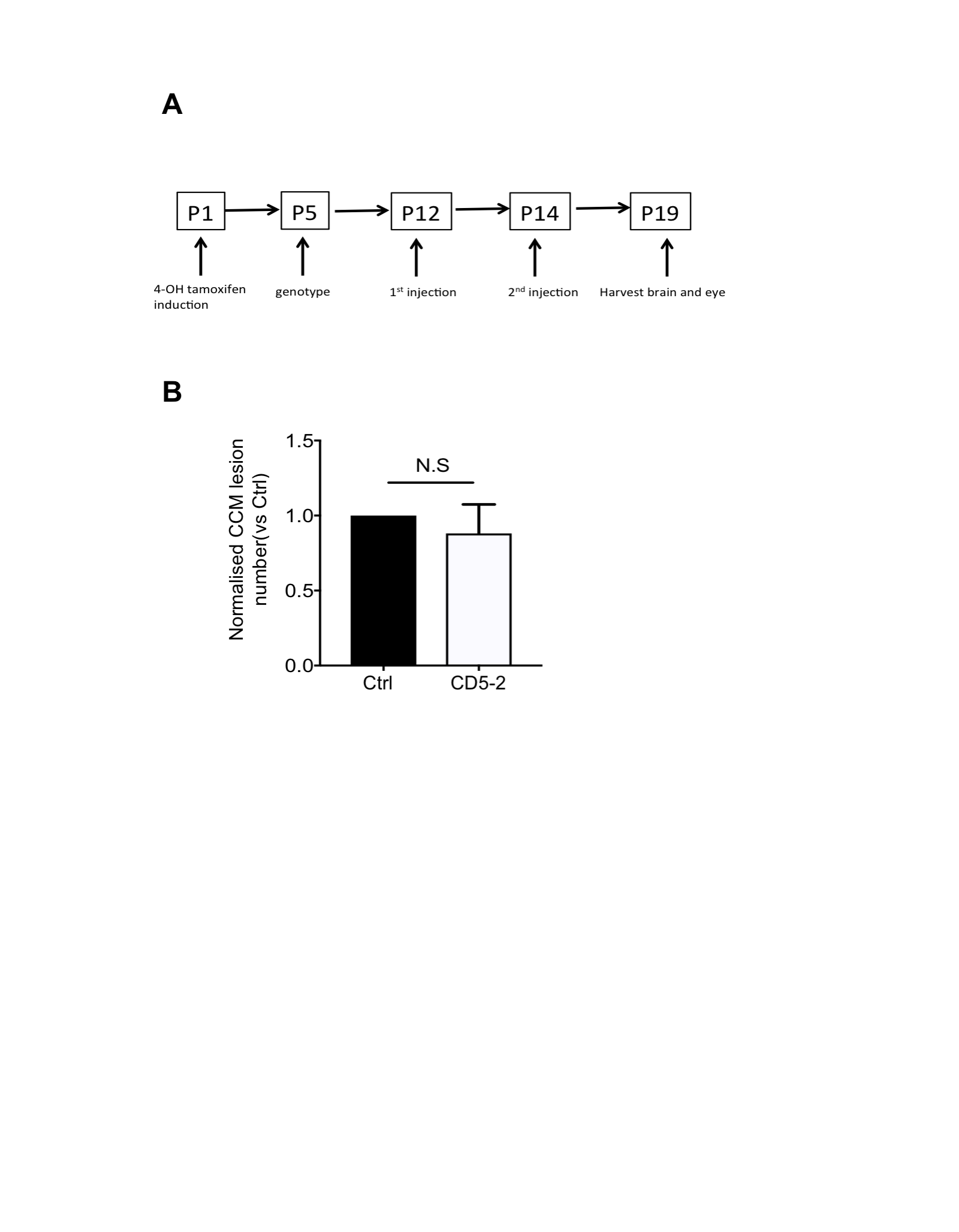

Supplement: S5 Fig — (A) Experimental protocol for late stage treatment: Ctrl or CD5-2 (30 mg/kg) was administered by IP injection at P12 and P14, and brains and retinas were dissected at P19. (B) Number of lesions from mice treated in (A) (n = 3, from 3 litters). Values are shown as mean ± SEM. N.S, not significant, determined by Student t test. For the raw data used for quantification, see S5 Fig in S1 Data. CCM, cerebral cavernous malformation; Ctrl, control; IP, intraperitoneal (TIF) [file pbio.3000734.s005.tif]

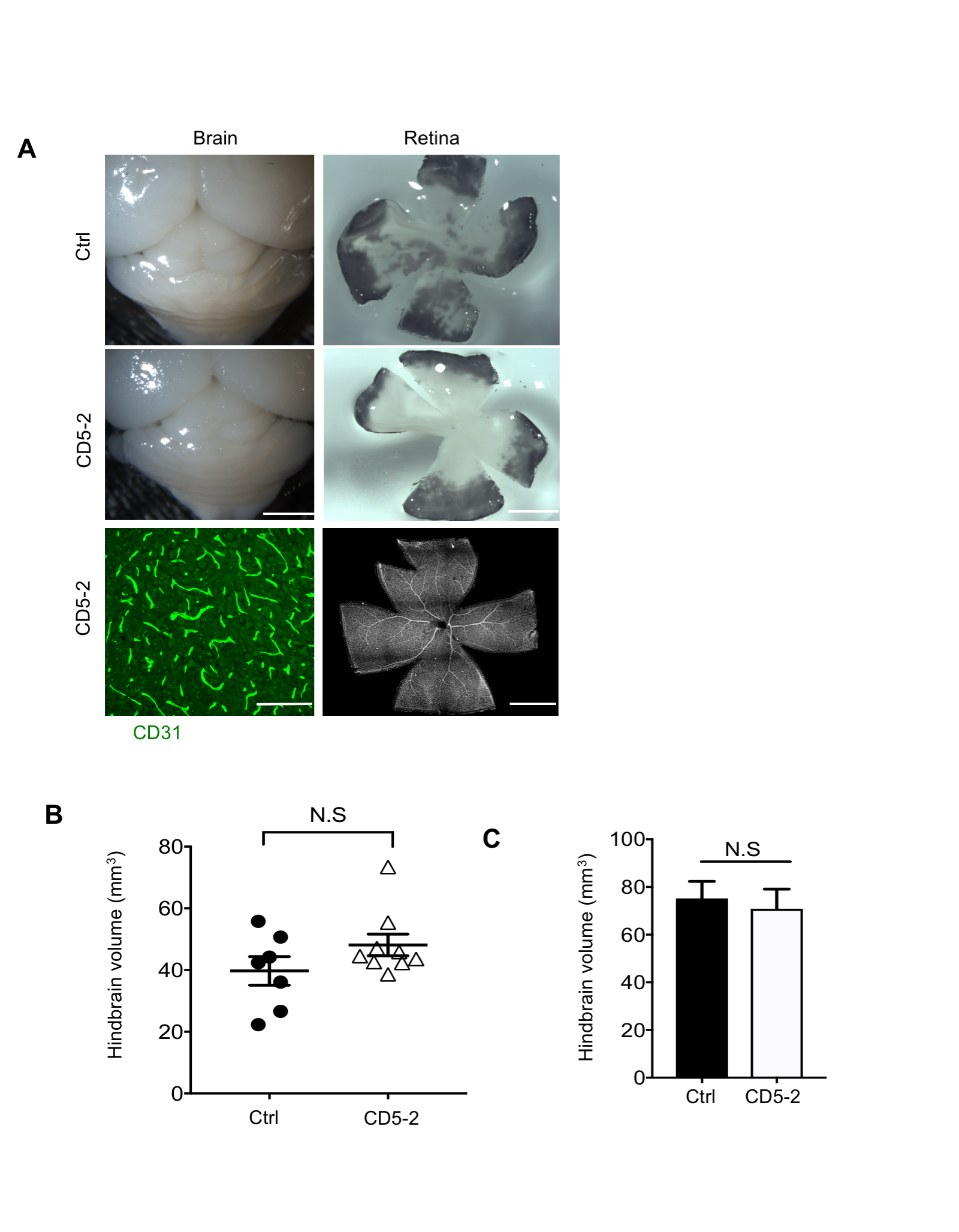

Supplement: S6 Fig — (A) Representative images of hindbrain, retina, and their vasculature from Ctrl- and CD5-2–treated WT mice. Bars, brain, 1 mm; retina, 200 μm; bottom panel left, 100 μm; right, 200 μm. Samples were dissected at P12. (B) Treatment of CD5-2 has no effect on size of hindbrain from mice treated at P6 and P8; n = 7 mice for control-treated group and n = 9 mice for CD5-2–treated group. Mice are from 4 different litters. (C) Treatment of CD5-2 has no effect on size of hindbrain from mice treated at P12 and P14 compared with Ctrl-treated mice (n = 4, from 4 litters). Values are shown as mean ± SEM. N.S, not significant, determined by Student t test. For the raw data used for quantification, see S6 Fig in S1 Data. CCM, cerebral cavernous malformation; Ctrl, control; WT, wild-type (TIF) [file pbio.3000734.s006.tif]

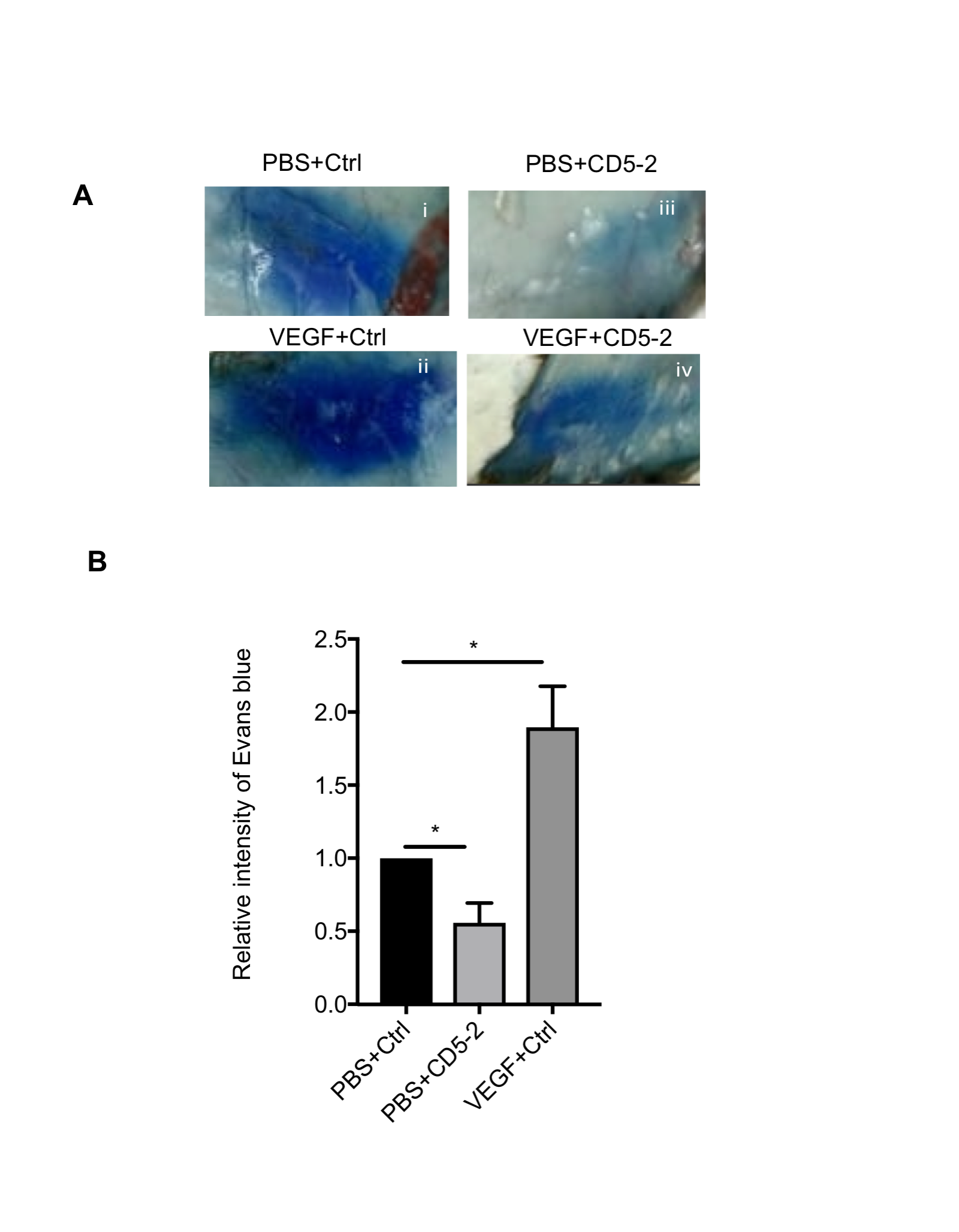

Supplement: S7 Fig — (A) Miles assay of dermal permeability in heterozygous Ccm1 (Tie2-Cre, Ccm1 fl/+) mice in response to PBS or VEGF after pretreatment with Ctrl or CD5-2. (B) Quantification of Evans blue extravasation, three mice for each group. Bar, 500 μm. Values are shown as mean ± SEM. *P < 0.05, determined by one-way ANOVA with Tukey correction. For the raw data used for quantification, see S7 Fig in S1 Data. CCM, cerebral cavernous malformation; Ctrl, control; VEGF, vascular endothelial growth factor. (TIF) [file pbio.3000734.s007.tif]

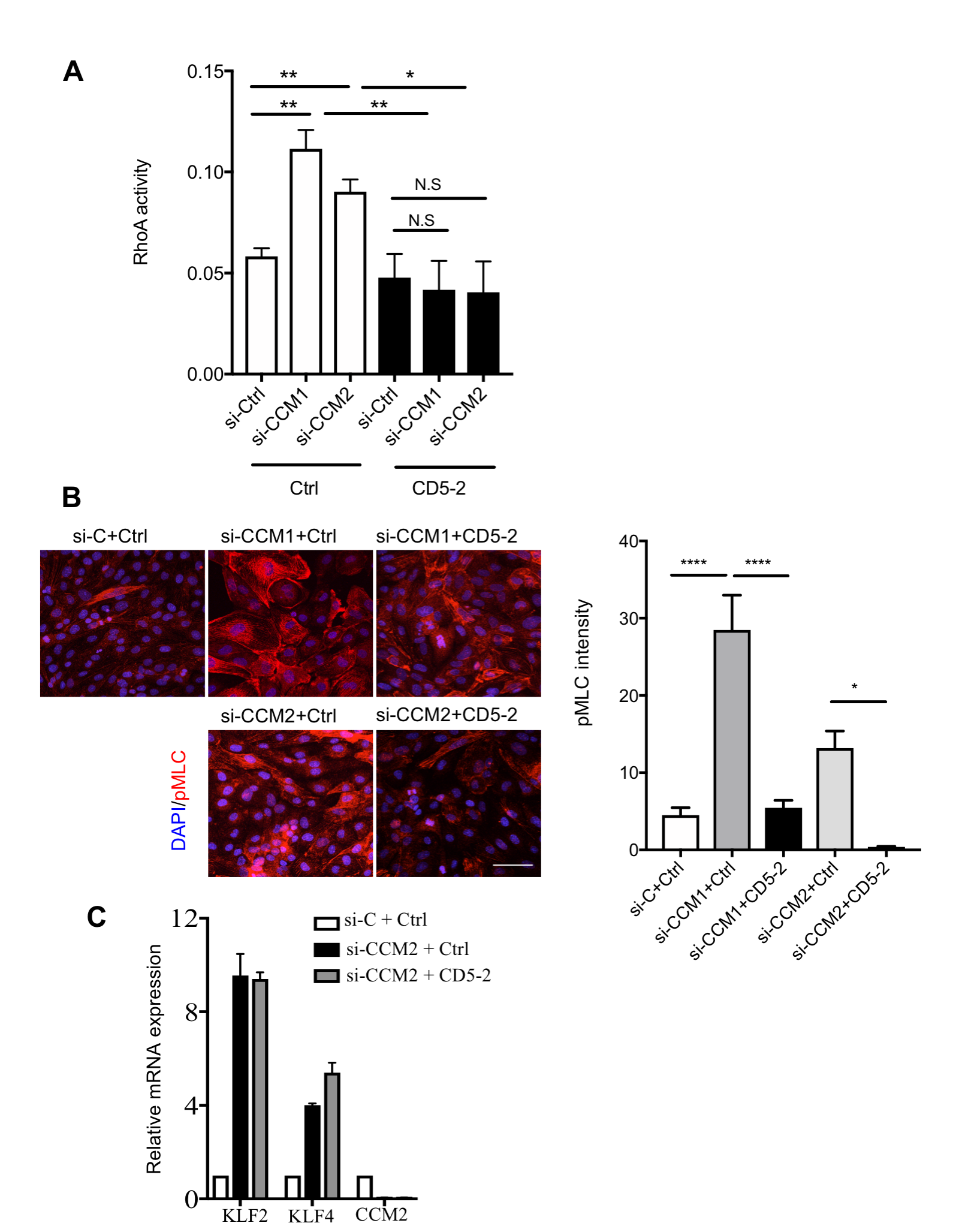

Supplement: S8 Fig — HUVECs were treated with 10 nM siRNAs for scramble control, CCM1, or CCM2 (si-Ctrl, si-CCM1, or si-CCM2) for 4 hours, followed by transfection of 15 nM CD5-2 or Ctrl, then cultured for 48 hours. (A) RhoA activity was measured using RhoA G-LISA activation assay kit (n = 4). (B) Immunostaining was performed to measure pMLC. Representative images are shown. Intensity of staining was quantified from 3–5 images per treatment, n = 3 experiments. Bar, 60 μm. Quantification of immunoblots was by Fiji ImageJ software (n = 3–5). (C) mRNA levels of KLF2 and KLF4 following Ctrl or CD5-2 treatment in CCM1- or CCM2-depleted ECs, analyzed by real-time PCR analysis (n = 2). Data represent mean ± SEM. *P < 0.05, **P < 0.01, ****P < 0.001, determined by one-way ANOVA with Tukey correction. For the raw data used for quantification, see S8 Fig in S1 Data. CCM, cerebral cavernous malformation; Ctrl, control; EC, endothelial cell; G-LISA, G protein linked immunosorbent assay; HUVEC, human umbilical vein endothelial cell; KLF2/4, kruppel-like factor 2/4; pMLC, phospho-myosin light chain; RhoA, ras homologue A; ROCK, Rho-associated protein kinase; siRNA, small interfering RNA (TIF) [file pbio.3000734.s008.tif]

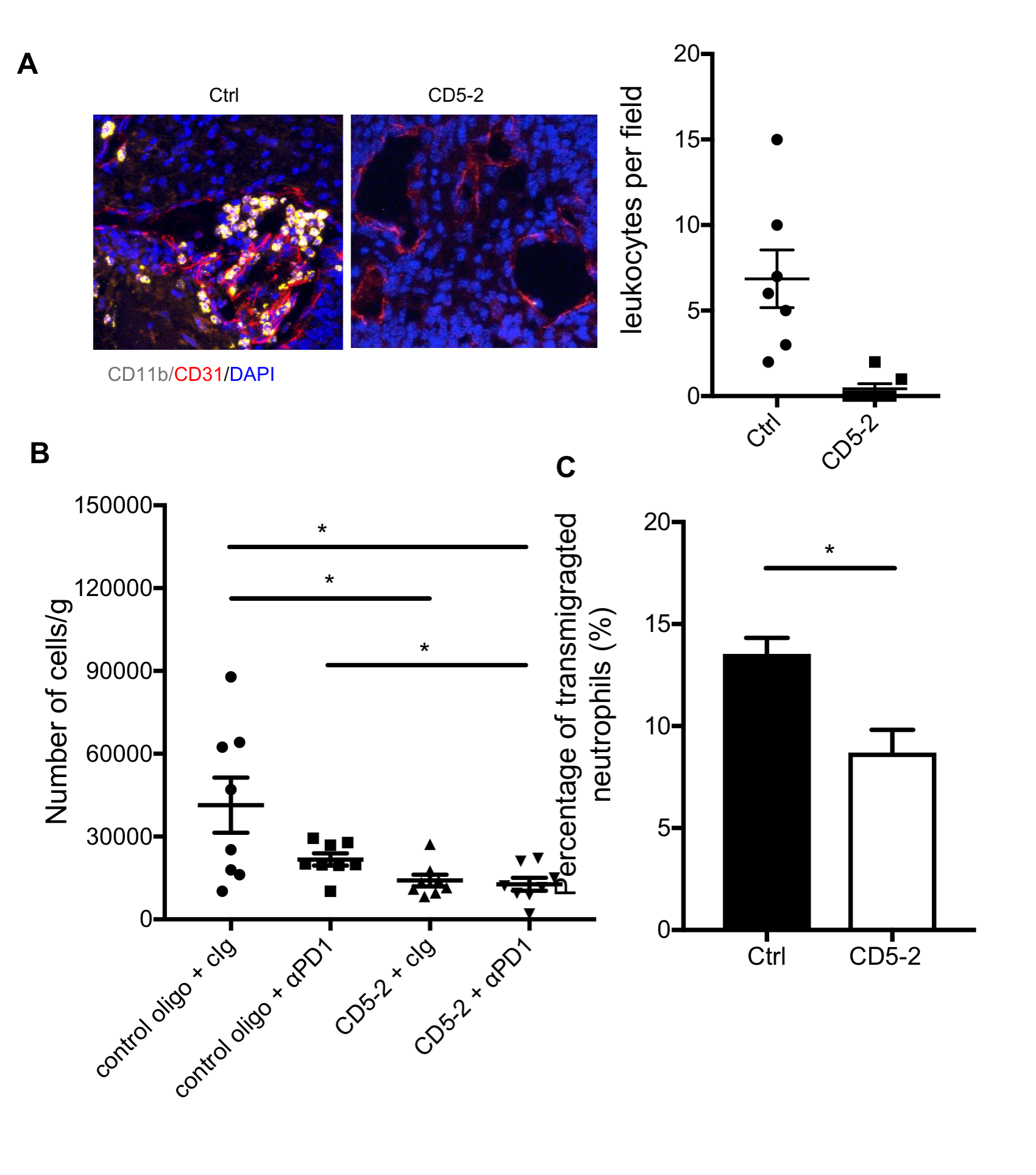

Supplement: S9 Fig — (A) CD11b+ (yellow) leukocytes accumulate in CCM lesions treated with CD5-2. Blood vessels are stained with CD31 (red), and cell nuclei are stained with DAPI (blue). Images in all panels are representative of at least 3 mice in each group. Quantification of number of CD11b+ cells in CCM lesion. (B) Quantification of number of CD11b+Gr1hi cells in MC38 tumor model (n = 8). (C) Quantification of percentage of transmigrated neutrophils (%) across the control- and CD5-2–treated ECs in the transwell migration assay (n = 3). For the raw data used for quantification, see S9 Fig in S1 Data. CCM, cerebral cavernous malformation; EC, endothelial cell (TIF) [file pbio.3000734.s009.tif]

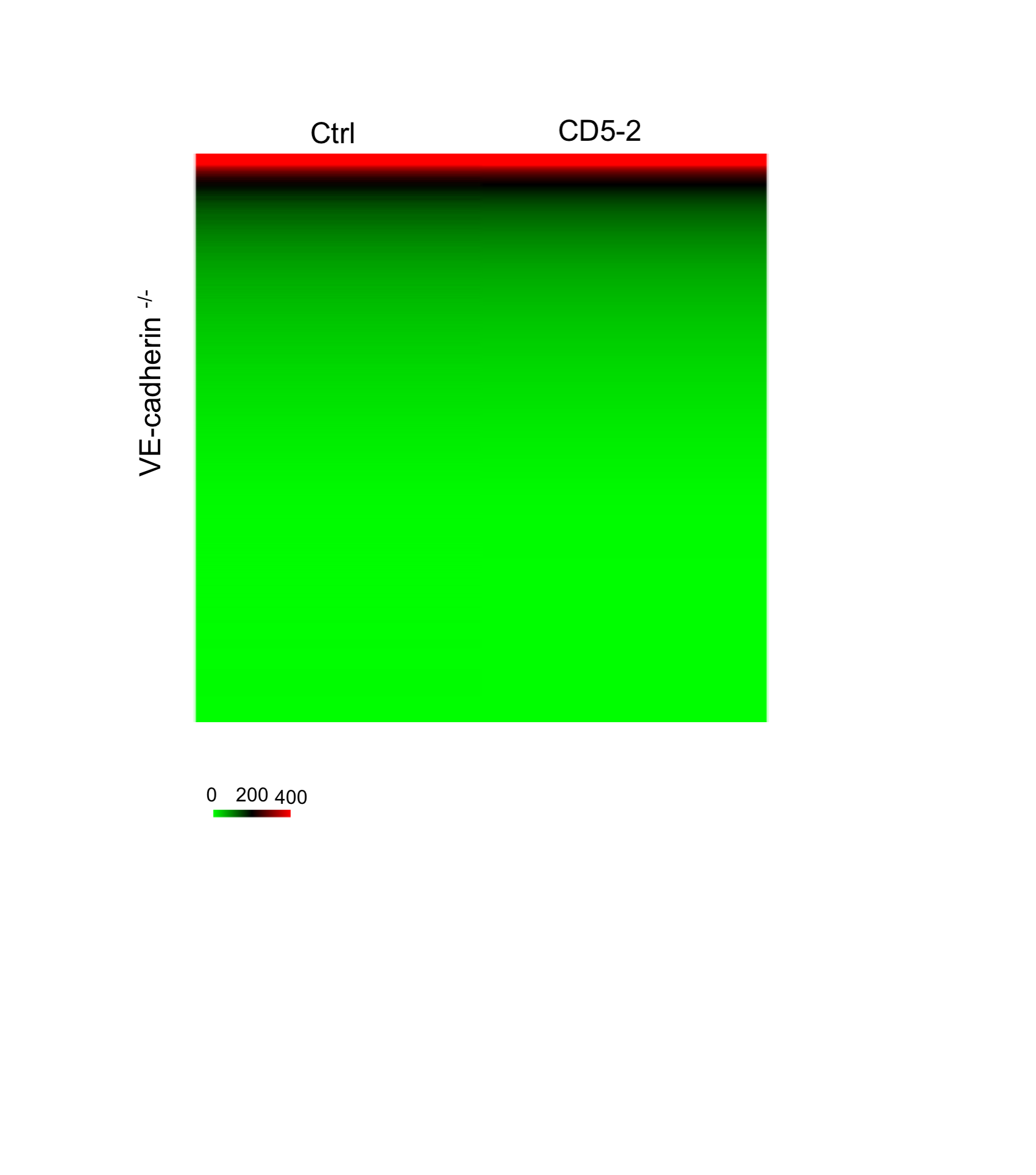

Supplement: S10 Fig — Heatmap of all genes between Ctrl- and CD5-2–treated VE-cadherin null ECs (VE-cadherin−/−). Ctrl, control; EC, endothelial cell; HUVEC, human umbilical vein endothelial cell; VE-cadherin, vascular endothelial cadherin (TIF) [file pbio.3000734.s010.tif]

## Slide 1
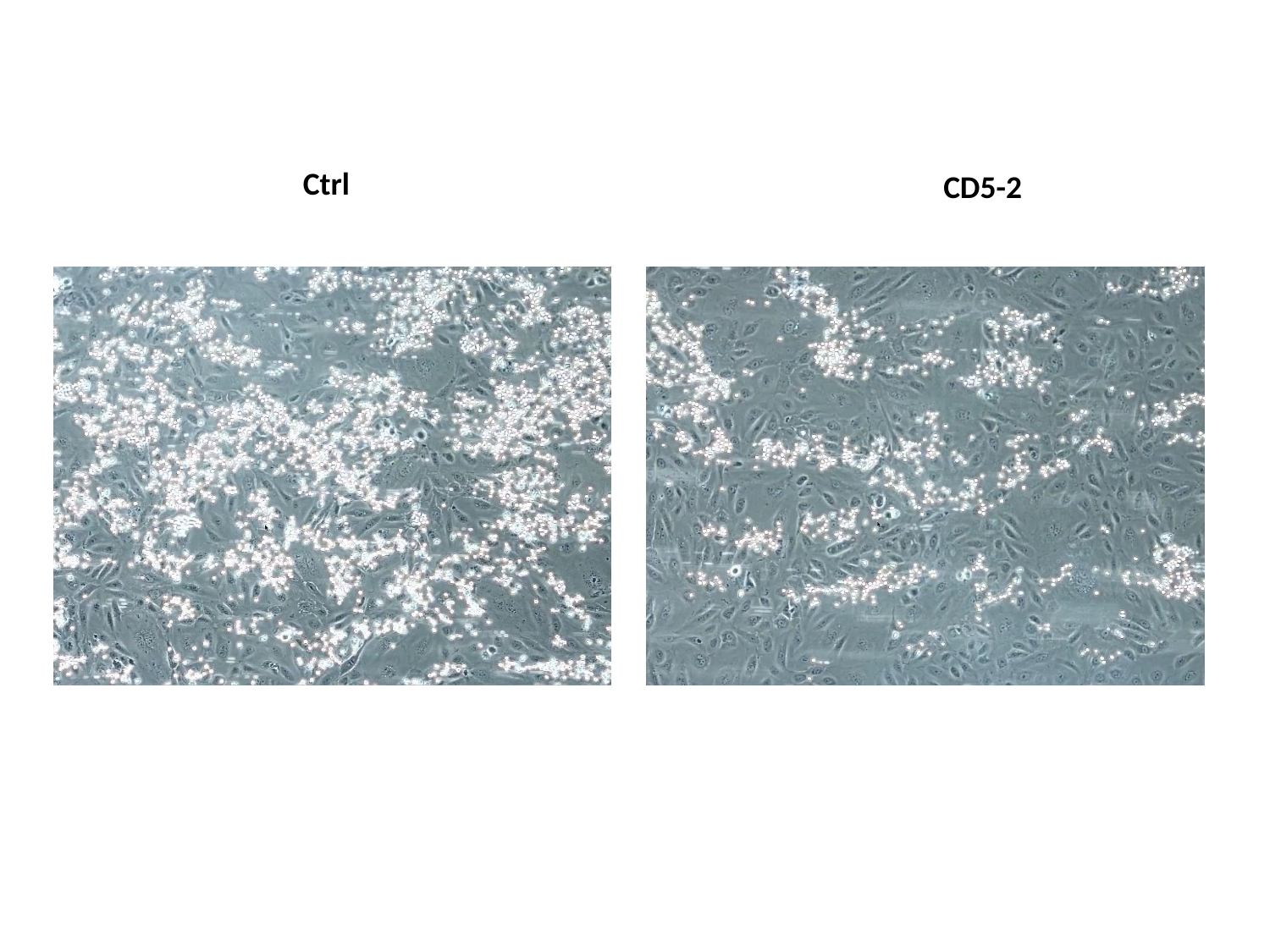

Ctrl
CD5-2

Supplement: S1 Movie — Dynamic adhesion of neutrophils onto TNF-α–stimulated EC monolayer treated with control and CD5-2. HUVECs treated with control and CD5-2 were plated onto the μ-Slide I Luer (Ibidi, Germany). The neutrophils in HUVEC medium were added into the ibidi pump system (Ibidi, Germany). The dynamic adhesion of neutrophils onto control- or CD5-2–treated HUVECs was recorded under an inverted phase-contrast microscope after 6 minutes of rolling. EC, endothelial cell; HUVEC, human umbilical vein endothelial cell; TNF-α, tumor necrosis factor-α (PPTX) [file pbio.3000734.s011.pptx]

## Slide 1
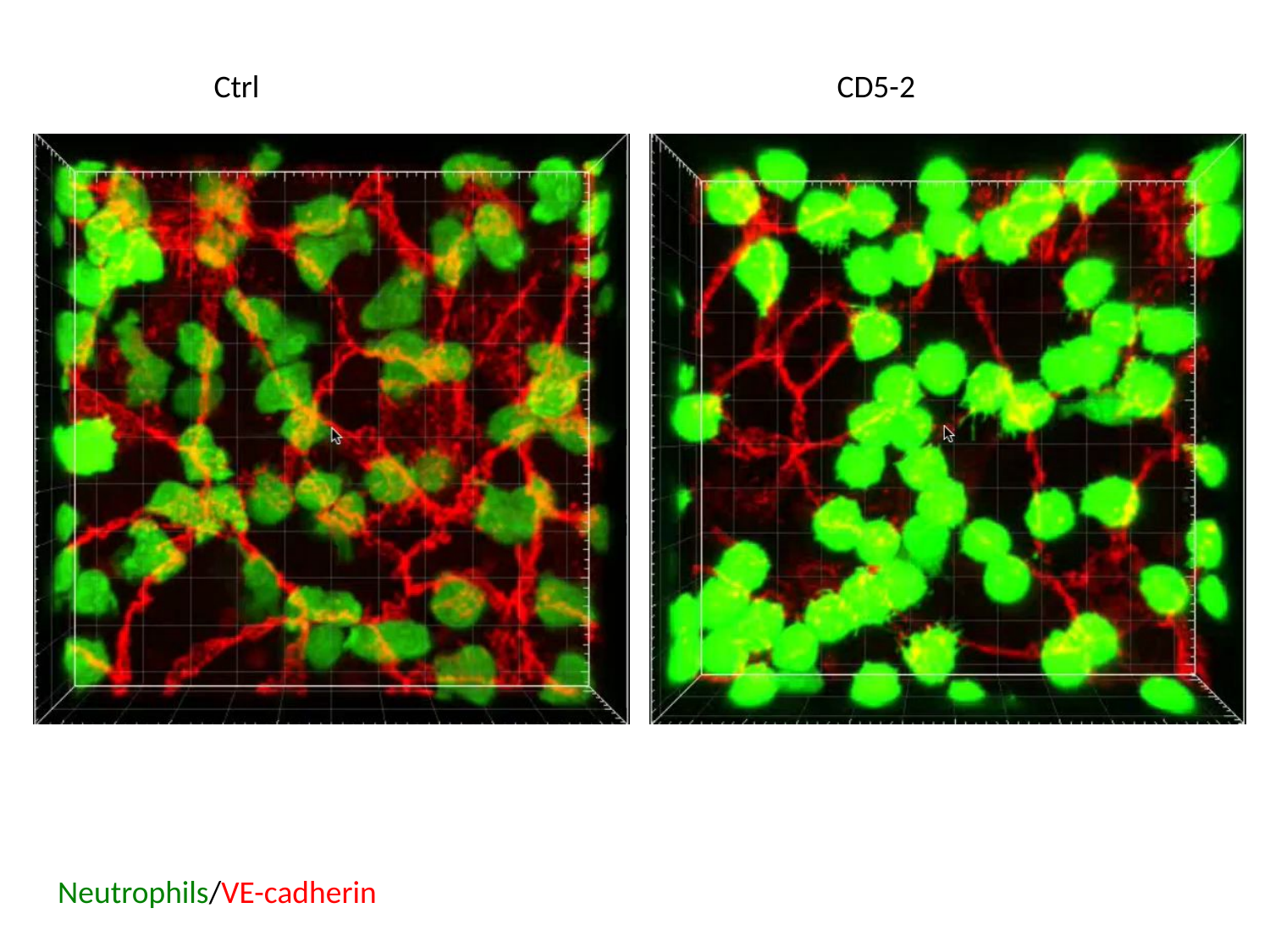

Ctrl
CD5-2
Neutrophils/VE-cadherin

Supplement: S2 Movie — Paracellular transmigration of GFP-labeled neutrophils (green) through TNF-α–stimulated endothelium (VE-Cadherin, red) in the presence of control and CD5-2 in vitro. The majority of neutrophils treated with control transmigrated via the paracellular route, as assessed in real time, by contact and passage through the area bonded by VE-cadherin (red), whereas CD5-2 inhibited neutrophil transmigration. GFP, green fluorescent protein; TNF-α, tumor necrosis factor α; VE-cadherin, vascular endothelial cadherin (PPTX) [file pbio.3000734.s012.pptx]

## Slide 1
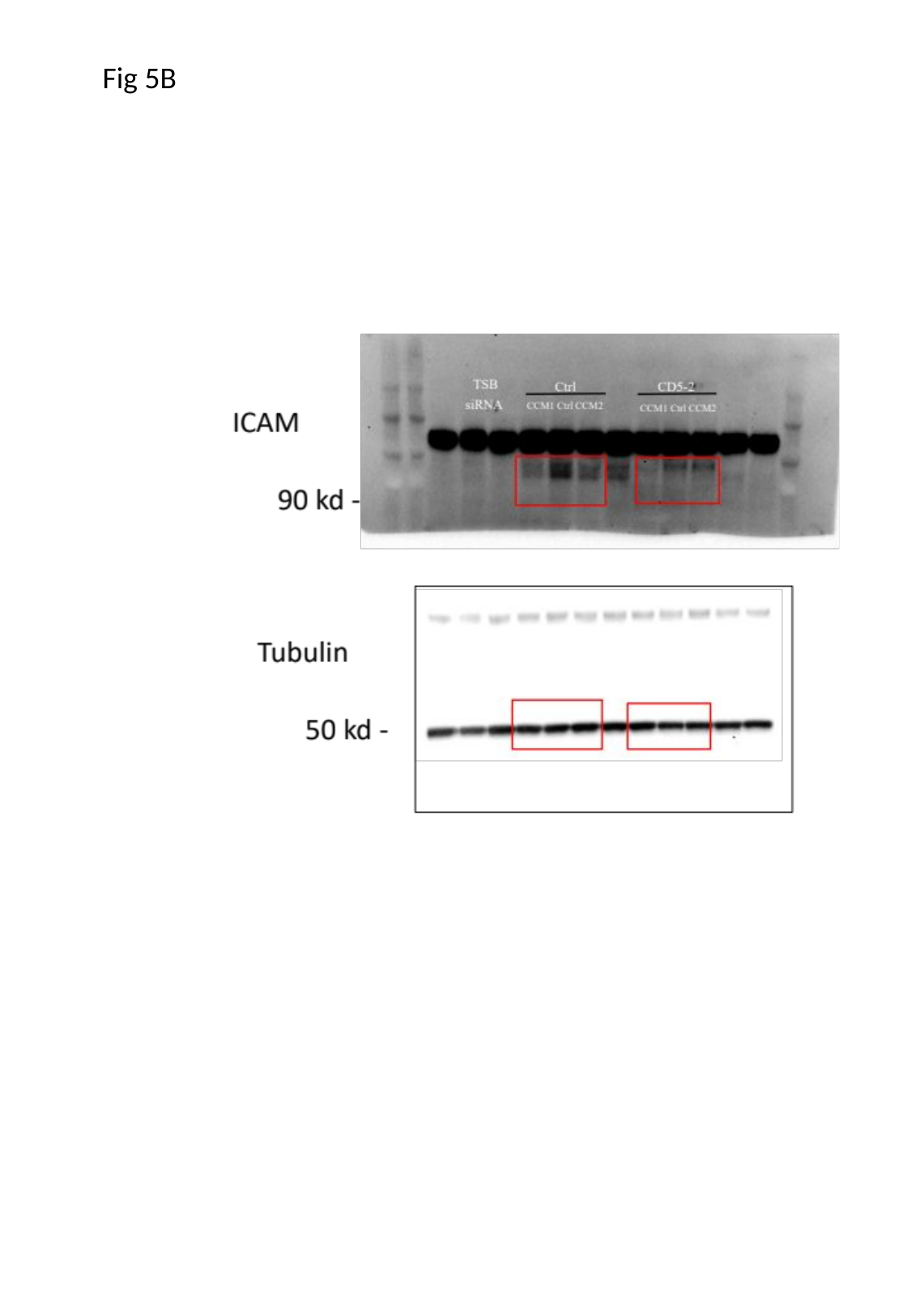

Fig 5B

## Slide 2
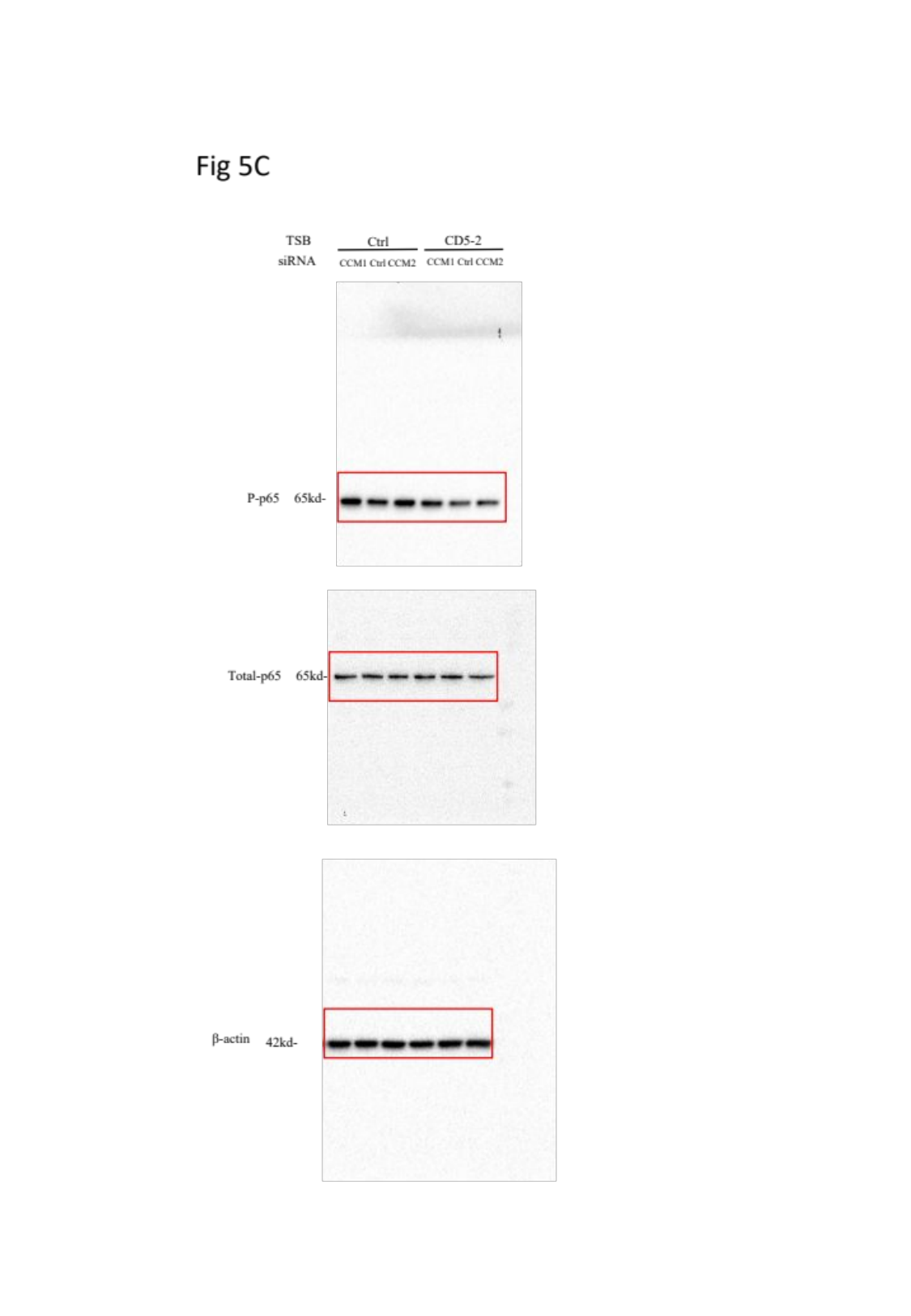

## Slide 3
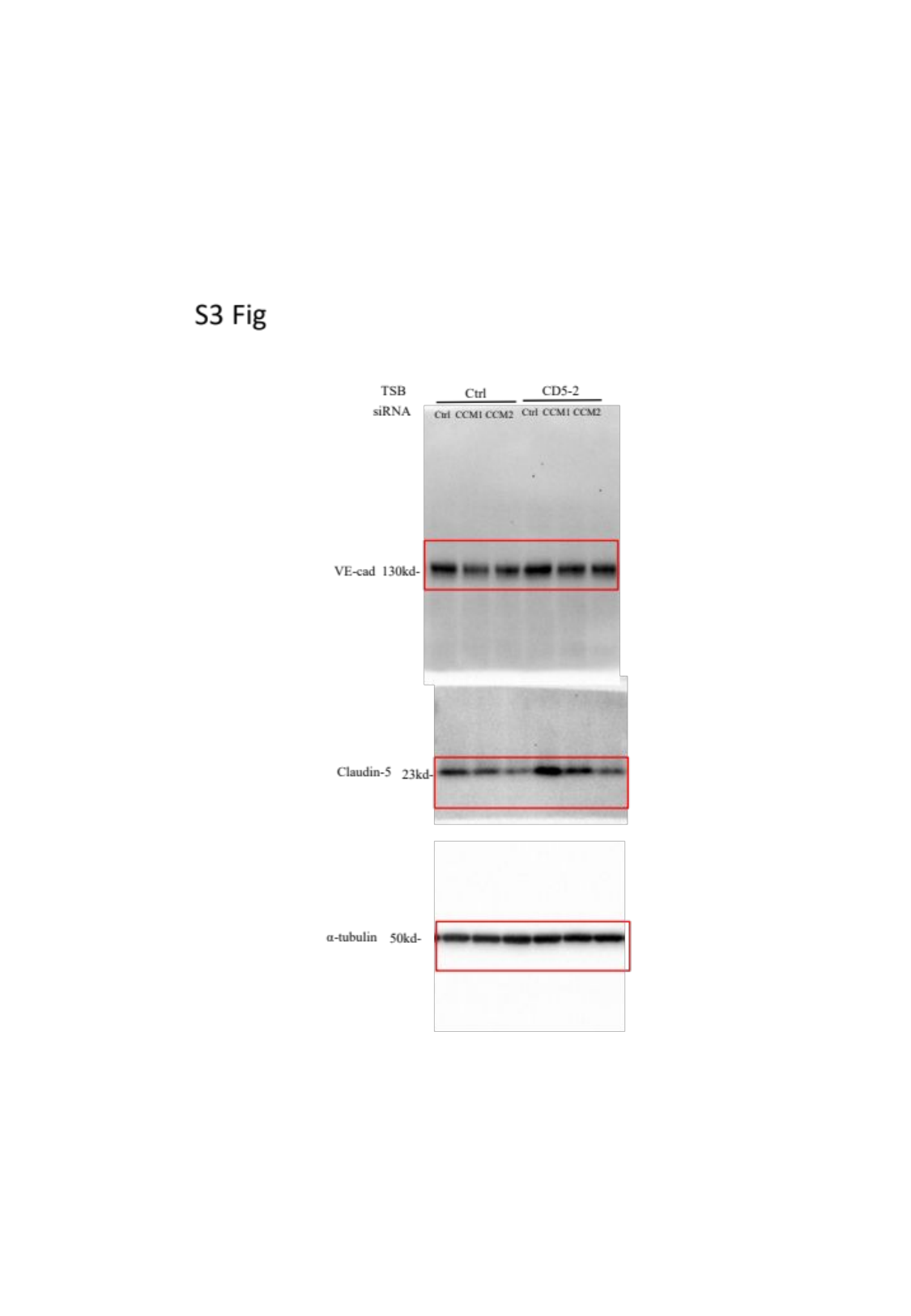

Supplement: S1 Raw images — (PPTX) [file pbio.3000734.s014.pptx]
